# Anastasis enhances metastasis and chemoresistance of colorectal cancer cells through upregulating cIAP2/NFκB signaling

**DOI:** 10.1038/s41419-023-05916-8

**Published:** 2023-06-30

**Authors:** Ru Wang, Yuxing Wang, Xiaohe Liu, Menghao Liu, Lili Sun, Xiaohua Pan, Huili Hu, Baichun Jiang, Yongxin Zou, Qiao Liu, Yaoqin Gong, Molin Wang, Gongping Sun

**Affiliations:** 1 Key Laboratory of Experimental Teratology, Ministry of Education, Institute of Molecular Medicine and Genetics, School of Basic Medical Sciences, Cheeloo College of Medicine, Shandong University, Jinan, Shandong 250012 China; 2 Key Laboratory of Experimental Teratology, Ministry of Education, Department of Histology and Embryology, School of Basic Medical Sciences, Cheeloo College of Medicine, Shandong University, Jinan, Shandong 250012 China; 3 Department of Breast and Thyroid Surgery, Shandong Provincial Hospital Affiliated to Shandong First Medical University, Jinan, Shandong 250021 China; 4 Department of Systems Biomedicine and Research Center of Stem Cell and Regenerative Medicine, School of Basic Medical Sciences, Cheeloo College of Medicine, Shandong University, Jinan, Shandong 250012 China

**Keywords:** Colorectal cancer, Metastasis

## Abstract

Chemotherapy is a common strategy to treat cancer. However, acquired resistance and metastasis are the major obstacles to successful treatment. Anastasis is a process by which cells survive executioner caspase activation when facing apoptotic stress. Here we demonstrate that colorectal cancer cells can undergo anastasis after transient exposure to chemotherapeutic drugs. Using a lineage tracing system to label and isolate cells that have experienced executioner caspase activation in response to drug treatment, we show that anastasis grants colorectal cancer cells enhanced migration, metastasis, and chemoresistance. Mechanistically, treatment with chemotherapeutic drugs induces upregulated expression of cIAP2 and activation of NFκB, which are required for cells to survive executioner caspase activation. The elevated cIAP2/NFκB signaling persists in anastatic cancer cells to promote migration and chemoresistance. Our study unveils that cIAP2/NFκB-dependent anastasis promotes acquired resistance and metastasis after chemotherapy.

## Introduction

Colorectal cancer is the third most common cancer in the world and ranks second in terms of mortality [1, 2]. Chemotherapy, which aims at killing cancer cells, is a common strategy for colorectal cancer treatment [3, 4]. However, some cancer cells manage to survive chemotherapy and propagate, leading to cancer relapse and metastasis [5–7]. Although numerous mechanisms, including changes in drug metabolism, aberrant gene regulation, cancer stem cells, and alterations of cell death program, have been reported to contribute to chemotherapy resistance [8–10], the underlying mechanisms remain unclear.

An important strategy by which chemotherapy eliminates cancer cells is apoptosis. Caspases, a group of cysteine proteases, are key mediators of apoptosis. Activation of executioner caspases, which leads to cleavage of diverse substrates and eventually results in cell dismantlment, was once considered “a point of no return” in the process of apoptosis [11–13]. In recent years, accumulating evidence has demonstrated that cells can survive apoptotic stress even after executioner caspase activation through a process named anastasis [14–16]. Anastasis or survival from stress-induced executioner caspase activation has been reported in a group of mammalian cell lines after exposure to chemical stress like ethanol, staurosporine, death receptor ligands, and chemotherapeutic drugs [14, 17–22]. It also occurs in vivo in epithelial tissues after wounding [23]. Studies on breast cancer cells, melanoma cells, cervical cancer cells, and ovarian cancer cells have shown that anastasis grants cancer cells some new features [17–22]. For example, anastatic breast cancer cells and cervical cancer cells exhibit increased drug resistance and migration [21]. Melanoma cells that survive executioner caspase activation induced by transient tBid overexpression or exposure to chemotherapeutic drug dacarbazine display elevated in vitro cell migration and in vivo metastasis [20].

In this study, using a lineage tracing system to label and isolate cells that have experienced executioner caspase activation and their descendants, we demonstrate anastatic colorectal cancer cells acquire enhanced migration, metastasis, and chemoresistance through elevated cIAP2 expression and NFκB activity. Furthermore, we show that both NFκB activation and cIAP2 expression are induced by chemotherapeutic drugs and are essential for anastasis.

## Materials and methods

### Cell culture

Human colorectal cancer cell lines HCT-116 (Cat# TCHu99) and HT-29 (Cat# TCHu103) were purchased from Cell Bank of the Chinese Academy of Sciences (Shanghai, China), and cultured in RPMI-1640 (Cat# C11875500BT, Gibco, BRL, Grand Island, NY, USA) supplemented with 10% fetal bovine serum (Cat# 60211031, SuperCulture, Shenzhen, China). Cells were maintained in a humidified atmosphere at 37 °C with 5% CO_2_ and routinely tested for Mycoplasma. When evaluating the effects of different treatments or gene manipulations, cells were randomly allocated into different groups.

### Animal experiments

All animal experiments were performed in accordance with protocols approved by the Institutional Animal Care and Use Committee, School of Basic Medical Sciences, Shandong University. All mice were housed in a pathogen-free animal facility. To evaluate the cancer cell colonization in lungs, 1 × 10^6^ cells in 100 μL PBS were injected into six-week-old male BALB/c nude mice (Beijing Vital River Laboratory Animal Technology, Cat# 401, Beijing, China) through the tail veins. To evaluate liver metastasis after splenic injection, 2 × 10^6^ cells in 100 μL PBS were injected into the distal spleen of seven-week-old female nude mice. 2 × 10^6^ cells in 50 μL PBS were injected into cecum of seven-week-old female nude mice to generate orthotopic tumors according to the published protocol [24].

For the in vivo chemoresistance model, 1 × 10^6^ cells in 100 μL PBS were subcutaneously injected into the flank of six-week-old male BALB/c nude mice. The tumors were measured every 3 days and tumor volumes were calculated by the formula: volume = width^2^ × length/2. When the tumors became palpable (50–100 mm^3^), irinotecan (Cat# HY-16562, MedChemExpress, Shanghai, China) was administered by intraperitoneal injection at 40 mg/kg twice a week. After six injections, the mice were sacrificed and the tumors were collected and imaged.

### Plasmids

To generate pLKO.1-BIRC3 shRNA plasmid, the shRNA targeting *BIRC3* (CCTGGATAGTCTACTAACTTTCAAGAGAAGTTAGTAGTAGACTATCCAGGTTTTT) was inserted into pLKO.1 vector (Cat# 20211013001, WZ Biosciences, Jinan, China) between EcoRI site and SacII site. To make *BIRC3-*overexpressing construct, the coding sequence for *BIRC3* was cloned by PCR using cDNA from HCT-116 cells as template and inserted into pLV-IRES-BSD vector (Cat# 20220805038, WZ Bioscience) between XbaI site and BamHI site. pCW57-Lyn11-NES-DEVD-flpO-hygro and pCDH-FRT-STOP-FRT-ZsGreen-puro were reported previously [22]. pCDH-puro-CMV-GC3AI was obtained from Addgene (Cat# 78910).

### Lentivirus production and infection

pCDH-puro-CMV-GC3AI, pCDH-FRT-STOP-FRT-ZsGreen-puro, pCW57-Lyn11-NES-DEVD-flpO-hygro, pLVX-BIRC3 shRNA or pLV-BIRC3 was transfected into HEK293T cells together with pCMV-dR8.2 dvpr (Addgene# 8455) and pCMV-VSV-G (Addgene# 8454) using Lipofectamine 2000 (Cat# 11668019, Invitrogen, New York, USA) to produce lentivirus carrying *GC3AI*, *Lyn11-NES-DEVD-FLP*, *FRT-STOP-FRT-ZsGreen*, *BIRC3 shRNA* or *BIRC3*. The supernatant was harvested and filtered with a 0.45 μm filter at 48 h and 72 h post transfection. Colorectal cancer cells were infected overnight in the presence of 10 μg/mL polybrene (Cat# H8761, Solarbio, Beijing, China) and then selected for 5–7 days in growth medium containing 2 μg/mL puromycin (Cat# P8230, Solarbio), 250 μg/mL hygromycin (Cat# H8081, Solarbio) or 10 μg/ml Blasticidin S (Cat# B9300, Solarbio).

### Isolation of the ZsGreen^+^, ZsGreen^−^ and control populations

HCT-116^CasE^ cells were treated with 20 nM paclitaxel (Cat# HY-B0015, MedChemExpress) for 24 h or with 0.5 μM 5′-fluorouracil (Cat# HY-90006, MedChemExpress) for 12 h. HT-29^CasE^ cells were treated with 10 nM paclitaxel for 24 h. The treatment medium was then replaced with fresh growth medium to allow cells to recover. After 48 h recovery, cells were applied to fluorescence activating cell sorting (FACS) on Moflo Astrios EQ (Beckman Coulter, Brea, CA) to collect ZsGreen^+^ and ZsGreen^−^ cells. To obtain the control population, HCT-116^CasE^ or HT-29^CasE^ cells were treated with 0.1% DMSO for 24 h, recovered in regular growth medium for 48 h, then applied to FACS. 1 μg/mL doxycycline was added to cells 6 h before treatment with chemotherapeutic drugs or 0.1% DMSO and removed together with them. All the addition or removal of chemicals was accompanied by medium change.

### Annexin V-PI staining and flow cytometry analysis

At the end of drug treatment, ~1 × 10^5^ cells were harvested from tissue culture plates and centrifuged at 1500 rpm for 5 min at room temperature. Medium supernatant was removed and cells were washed once in PBS. Cells were then resuspended in 0.1 mL of cold binding buffer. 5 μL of Annexin V-APC and 10 μL of propidium iodide (PI) were added to cells. After 15 min incubation at room temperature in dark, cells were applied through cell strainer (70 μm, Cat# 352340, BD Biosciences, New York, USA) to remove aggregates. The stained cells were then analyzed by flow cytometry using CytoFLEX S. The data were processed using Flow Jo software (BD Biosciences).

### MTT assay

Cells were seeded in 96-well plates at 5 × 10^3^ cells per well. After overnight culture, cells were treated with chemotherapeutic drugs for 48 h. 20 μL of MTT reagent (Cat# HY-15924, MedChemExpress) was added to each well and the plate was incubated at 37 °C for 4 h. Absorbance at 492 nm was measured using Infinite M Nano 200 Pro (TECAN, Switzerland).

### Cell migration and invasion assays

Transwell assays were performed on 24-well plate with inserts (Cat# 353097, BD Biosciences) according to the manufacturer’s instructions. For cell migration assays, transwell plates were first incubated with serum-free medium at 37 °C for 30 min. For cell invasion assays, Matrigel (Cat# 354234, BD Biosciences) was diluted eight folds with cold medium. 50 μL diluted Matrigel was added to each insert. Then, 1 × 10^5^ cells in 200 μL serum-free RPMI-1640 (Gibco) were seeded into the inserts. 600 μL RPMI-1640 (Gibco) with 10% FBS was added in the lower chamber. 24 h later, cells that migrated or invaded through the pores were fixed in 4% paraformaldehyde for 20 min, stained with crystal violet, imaged on Axio Vert.A1 (Zeiss, Germany). The images were analyzed using ImageJ software (National Institutes of Health, USA).

### Hematoxylin and eosin staining

The tumors were fixed in 4% formaldehyde for 24 h. The fixed tissues were dehydrated and embedded in paraffin. Paraffin-embedded tumors were sliced into 4 μm sections, deparaffinized, and stained with Hematoxylin and Eosin.

### Quantitative RT-PCR

Total RNA was extracted with Trizol reagents (Cat# 15596026, ThermoFisher Scientific, New York, USA) following the manufacturer’s instructions. Total RNA (0.5–1 μg) was reverse transcribed into cDNA using Oligo (dT) primers and RevertAid RT kit (Cat# K1691, ThermoFisher Scientific). qPCR was done using Power SYBR Green PCR Master Mix (Cat# 4368706, ThermoFisher Scientific) on Light Cycler 480 System II (Roche Diagnostic, Basel, Schweiz). Actin was used as an internal control. All the primers used in this study were listed in Supplementary Table S1.

### Western blotting

Cells were lysed in RIPA buffer (Cat# 89901, ThermoFisher Scientific) supplemented with 1 mM proteinase inhibitor PMSF (Cat# KGP610, Keygen, Nanjing, China) and Phosphatase Inhibitor Cocktail (Cat# HY-K0013, MedChemExpress). Equal amounts of total protein were separated in 10% SDS-PAGE and transferred onto PVDF membrane. The membranes were blocked for 1 h at room temperature in TBST containing 5% BSA (Cat# A8020, Solarbio), and then incubated with primary antibodies overnight at 4 °C. After that, membranes were washed in TBST, incubated with secondary antibody conjugated with horseradish peroxidase for 1 h at room temperature. After washing with TBST, bands were detected using an enhanced chemiluminescence system (Cat# WB7108, ThermoFisher Scientific). All the antibodies used in this study are listed in Supplementary Table S2.

### RNA sequencing

Total RNA was extracted by Trizol reagent (Cat# 15596026, ThermoFisher Scientific) following the manufacturer’s instructions. RNA samples were processed using an Illumina Hiseq 2500 platform by Gene Denovo Biotechnology Co. (Guangzhou, China). Normalized count distributions were fit to a generalized linear model to test for differential expression of genes (*P* < 0.05) among multiple samples. Bioinformatic analyses were performed using online software provided by GENE DENOVO (https://www.omicsmart.com/home).

### siRNA transfection

2 × 10^6^ cells were plated in 10 cm Petri dishes 24 h prior to transfection. Cells were then transfected with 20 mM siRNA using TurboFect transfection reagent (Cat# 11668019, Invitrogen). The siRNAs used in this study are listed in Supplementary Table S3.

### Statistical analysis and reproducibility

Statistical analyses were performed using GraphPad Prism Version 7.0 (GraphPad Software, Inc.). Statistical significance was determined by two-tailed Student’s *t*-test for comparison between two samples and one-way ANOVA with Tukey test for comparison between multiple samples. The assumption of equal variance was validated by F-test. A *p* value <0.05 was considered statistically significant. The sample sizes were chosen empirically based on the observed effects and previous reports. The sample size for each experiment is listed in the figure legends. When collecting and analyzing data on RT-qPCR and xenograft volumes, the investigators were blinded to the group allocation. All the experiments except those involving mice were repeated at least three times. The experiments involving mice were repeated twice. All the repeats were successful, and the representatives were shown in the figures.

## Results

### Anastasis occurs in colorectal cancer cells after treatment with chemotherapeutic drugs

**Fig. 1:**
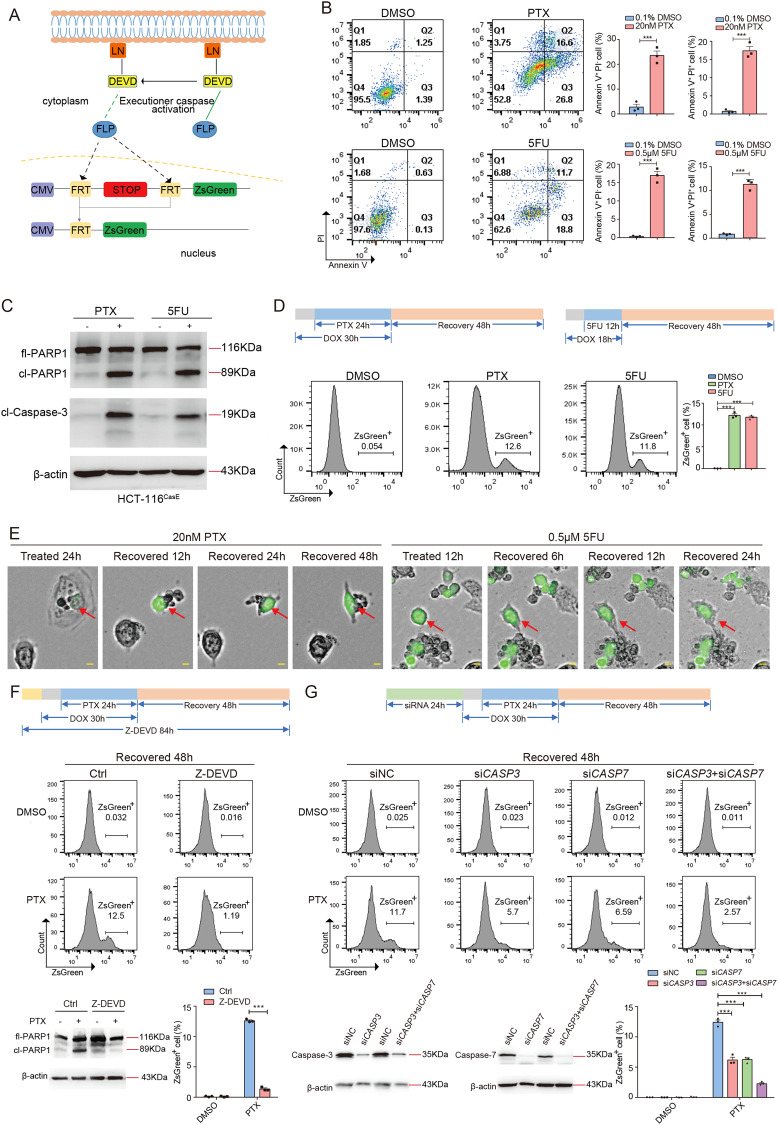
Anastasis occurs in colorectal cancer cells after treatment with chemotherapeutic drugs. **A** The schematic of mCasExpress sensor. LN: Lyn11-NES. **B** Annexin V - Propidium Iodide (PI) staining to detect apoptosis in HCT-116^CasE^ cells after 24 h treatment with 20 nM PTX or 0.1% DMSO (upper row) and after 12 h treatment with 0.5 μM 5FU or 0.1% DMSO (lower row). *N* = 3. **C** Western blots of full-length PARP1 (fl-PARP1), cleaved PARP1 (cl-PARP1), and cleaved caspase-3 (cl-Caspase-3) in HCT-116^CasE^ cells after 24 h treatment with 20 nM PTX or 0.1% DMSO (−), and after 12 h treatment with 0.5 μM 5FU or 0.1% DMSO (−). **D** Quantification of the percentage of ZsGreen^+^ cells in HCT-116^CasE^ cells after 48 h recovery from PTX or 5FU treatment by flow cytometry. *N* = 3. **E** Representative images from time-lapse live imaging of HCT-116^CasE^ cells during recovery after 24 h PTX treatment (left), and during recovery after 12 h 5FU treatment (right). The scale bars are 50 μm. The red arrows point to the examples of the cells undergoing anastasis. **F** Analysis of the effect of caspase inhibitor Z-DEVD-fmk (Z-DEVD) on the percentage of ZsGreen^+^ cells in HCT-116^CasE^ cells at 48 h recovery after PTX treatment. *N* = 3. The western blots show the efficiency of inhibition of executioner caspase activity through cleavage of caspase substrate PARP1. **G** Analysis of the effect of knocking down *CASP3* and/or *CASP7* on the percentage of ZsGreen^+^ cells in HCT-116^CasE^ cells at 48 h recovery after PTX treatment. *N* = 3. The western blots show the knockdown efficiency. In all bar graphs, error bars represent the standard error of the mean. *: *P* < 0.05. **: *P* < 0.01. ***: *P* < 0.001.

To investigate whether colorectal cancer cells can survive chemotherapy-induced executioner caspase activation, we generated HCT-116 cells carrying mCasExpress sensor (designated as HCT-116^CasE^). mCasExpress is comprised of an executioner caspase-activated DNA recombinase FLP (Lyn11-NES-DEVD-FLP) and an FLP activity reporter (FRT-STOP-FRT-ZsGreen) [22, 25] (Fig. 1A). mCasExpress can label cells that have experienced executioner caspase activation and their daughter cells with green fluorescence. The executioner caspase-activated FLP was expressed under the control of a doxycycline (DOX)-inducible promoter. Anastasis was defined as survival from executioner caspase activation in response to a transient stress that can kill the majority of cells when persists [15]. Paclitaxel (PTX) and 5’-fluorouracil (5FU) are commonly used chemotherapeutic drugs in clinic. Treatment with 20 nM PTX or 0.5 μM 5FU killed more than 90% of HCT-116^CasE^ cells within 96 h (Supplementary fig. S1A). Thus we first assessed whether HCT-116^CasE^ cells can undergo anastasis after transient exposure to 20 nM PTX or 0.5 μM 5FU. We treated HCT-116^CasE^ cells with PTX for 24 h or with 5FU for 12 h, then removed the drug to allow cells to recover. 24 h PTX treatment or 12 h 5FU treatment was sufficient to induce executioner caspase activation and apoptosis in HCT-116^CasE^ cells (Fig. 1B, C). After 48 h recovery, about 12% of the PTX or 5FU-treated cells were ZsGreen^+^, while in the control group, <0.1% cells were ZsGreen^+^ (Fig. 1D). Live imaging revealed some shrunk cells with green fluorescence gradually spread out and survived after removal of PTX or 5FU (Fig. 1E), indicative of ongoing anastasis. Treatment with caspase inhibitor Z-DEVD-fmk (Z-DEVD) or knocking down *CASP3* or/and *CASP7* significantly reduced the percentage of ZsGreen^+^ cells after 48 h recovery from PTX treatment (Fig. 1F, G), suggesting that the ZsGreen^+^ cells were cells that survived from PTX-induced executioner caspase activation. Anastasis was also observed in HT-29^CasE^ cells recovered after PTX treatment (Supplementary Fig. S1B–G) and HT-29^CasE^ and HCT-116^CasE^ cells recovered after treatment with two other chemotherapeutic drugs irinotecan and oxaliplatin (Supplementary Fig. S1H), indicating that anastasis may be a common response in colorectal cancer cells after treatment with chemotherapeutic drugs.

### Anastatic colorectal cancer cells acquire enhanced migration and metastasis

**Fig. 2:**
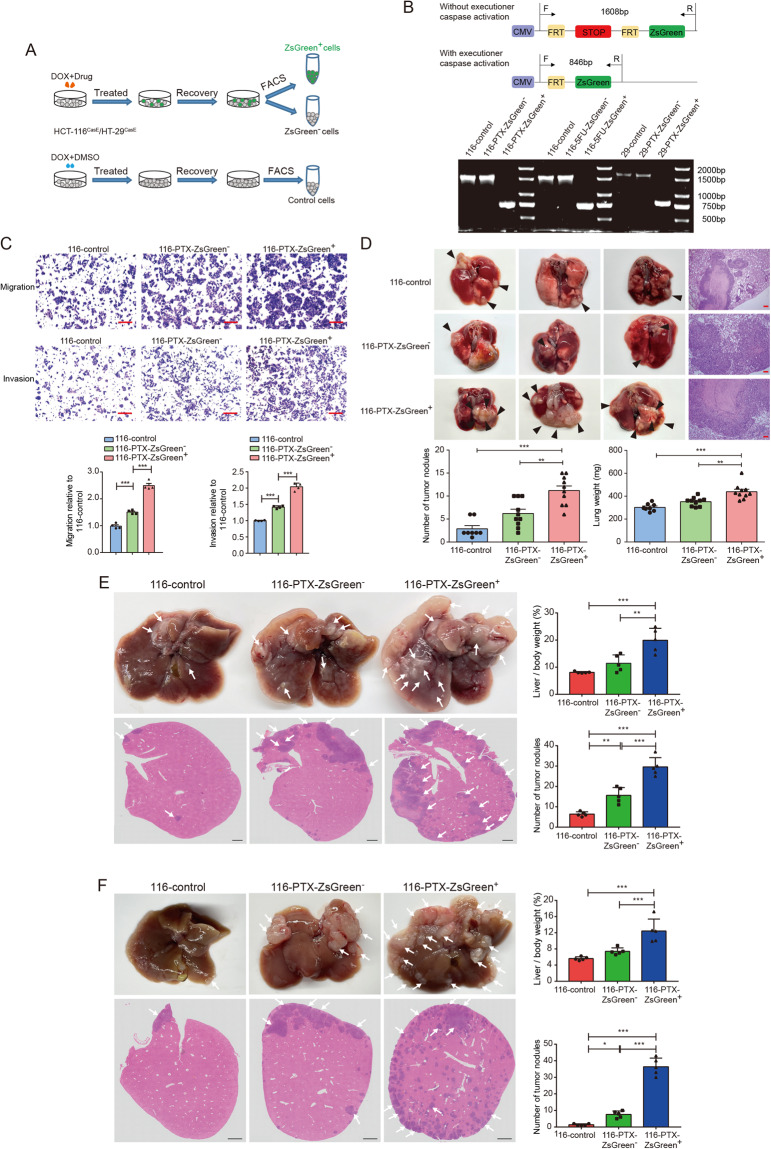
Anastatic colorectal cancer cells acquire enhanced migration and metastasis. **A** The workflow of isolating the ZsGreen^−^, the ZsGreen^+^, and the control populations. **B** Genotyping of different cell populations. The upper shows the position of primers and the size of PCR products. The lower is the result of genotyping. **C** Transwell assays to evaluate the migration and invasion capacity of 116-control, 116-PTX-ZsGreen^−^ and 116-PTX-ZsGreen^+^ cells. Scale bars are 50 μm. *N* = 4. **D** Lung colonization of 116-control, 116-PTX-ZsGreen^−^ and 116-PTX-ZsGreen^+^ cells. In the upper part are the images of representative lungs and Hematoxylin & Eosin (H&E) staining. Scale bars are 100 μm. The arrowheads point to examples of tumor nodules. In the lower row are the quantifications of the numbers of tumor nodules in lungs from each mice and the lung weights in mice injected with the indicated populations. *N* = 8 in 116 control group and *N* = 10 in 116-PTX-ZsGreen^−^ group and 116-PTX-ZsGreen^+^ group. **E** Liver metastasis of the indicated populations after splenic injection. Arrows in images of livers and H&E staining point to examples of tumor nodules. Scale bars are 1 mm. *N* = 5. **F** Liver metastasis of orthotopic tumors. Arrows in images of livers and H&E staining point to examples of tumor nodules. Scale bars are 1 mm. *N* = 5. In all bar graphs, error bars represent the standard error of the mean. *P < 0.05, **P < 0.01, ***P < 0.001.

To investigate whether anastasis grants colorectal cancer cells any new features, we isolated the ZsGreen^+^ populations and the ZsGreen^−^ populations from HCT-116^CasE^ and HT-29^CasE^ cells recovered from PTX or 5FU treatment and cultured them separately (designated as 116/29-PTX/5FU-ZsGreen^+^ and 116/29-PTX/5FU-ZsGreen^−^, respectively). The ZsGreen^−^ cells in HCT-116^CasE^ and HT-29^CasE^ cells recovered from control treatment (0.1% DMSO) were also isolated and cultured (designated as 116-control and 29-control, respectively) (Fig. 2A). All the populations can be cultured in vitro. Using primers flanking the FRT-STOP-FRT-ZsGreen cassette, we confirmed the purity of all the cell populations after long-term culture (Fig. 2B).

It has been reported that cervical cancer cells, melanoma cells, and breast cancer cells that survive executioner caspase activation acquire enhanced migration [17, 20, 21]. We tested whether migration and invasion of colorectal cancer cells are also affected by anastasis. Transwell assays showed that the ZsGreen^+^ populations were more migratory and invasive than the ZsGreen^−^ populations and the control populations (Fig. 2C, Supplementary Fig. S2A, B), suggesting the experience of executioner caspase activation increases migration and invasion of colorectal cancer cells.

We then investigated whether anastatic cells are more metastatic in vivo. We first injected the ZsGreen^+^ cells, the ZsGreen^−^ cells, and the control cells into nude mice through tail vein. After 45 days, more ZsGreen^+^ cells were colonized to the lungs than the ZsGreen^−^ and the control cells (Fig. 2D, Supplementary Fig. S2C, D). We also injected 116-PTX-ZsGreen^+^, 116-PTX-ZsGreen^−^ cells, and 116-control cells into the spleens of nude mice and harvested the livers after 2 weeks. Livers from mice injected with 116-PTX-ZsGreen^+^ cells had significantly higher tumor burden than those from mice injected with the other two cell populations (Fig. 2E). Furthermore, we injected 116-PTX-ZsGreen^+^, 116-PTX-ZsGreen^−^ and 116-control cells into the cecum of nude mice to generate orthotopic metastasis models. 116-PTX-ZsGreen^+^ cells exhibited significantly stronger liver metastasis than 116-PTX-ZsGreen^−^ and 116-control cells (Fig. 2F). These experiments together indicate that chemotherapy-induced anastasis enhances migration and metastasis of colorectal cancer cells.

### The enhanced migration and metastasis in anastatic cells are mediated by upregulation of *BIRC3*

**Fig. 3:**
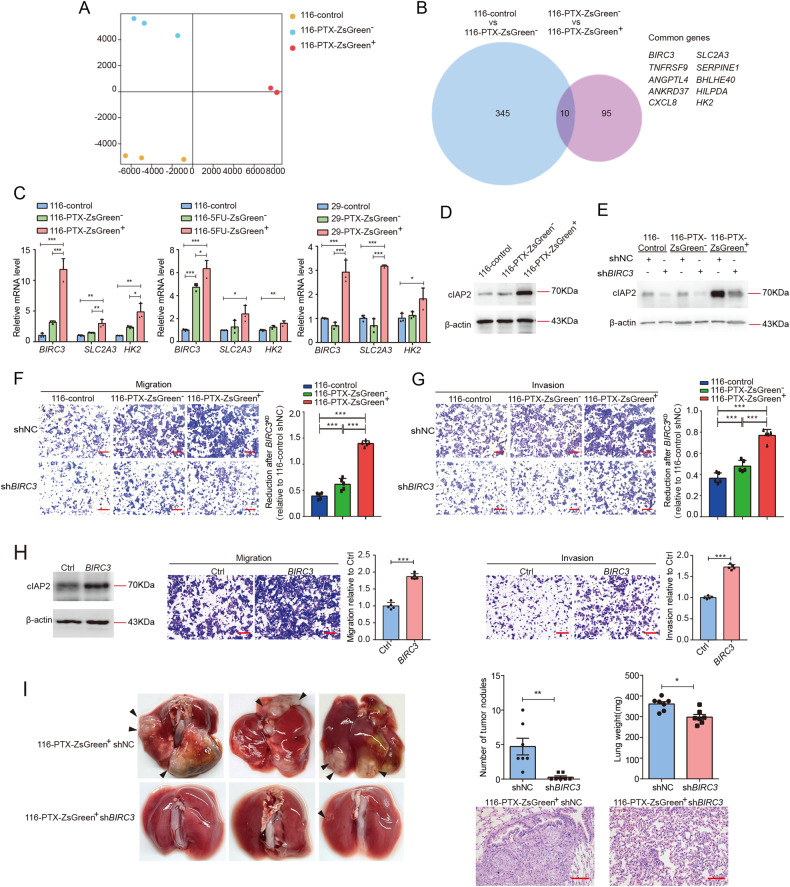
The enhanced migration and metastasis in anastatic cells are mediated by upregulation of *BIRC3*. **A** Principal component analysis of RNA sequencing data. *N* = 3. **B** The Venn diagram shows the overlap between the genes upregulated in 116-PTX-ZsGreen^−^ compared to 116-control and the genes upregulated in 116-PTX-ZsGreen^+^ compared to 116-PTX-ZsGreen^−^. **C** The mRNA expression of *BIRC3*, *SLC2A3,* and *HK2* in the indicated cells were determined by RT-qPCR. The data were normalized to the average expression in 116-control cells. *N* = 3. **D**, **E** The protein levels of cIAP2 in the indicated populations. **F**, **G** The effect of *BIRC3* knockdown on cell migration (**F**) and invasion (**G**). Scale bars are 50 μm. The bar graphs show reduction after knocking down *BIRC3* (*BIRC3*^*KD*^). Data were normalized to 116-control shNC. *N* = 5. **H** The effect of *BIRC3* overexpression on cell migration and invasion. *N* = 5. **I** The effect of knocking down *BIRC3* on lung metastasis of 116-PTX-ZsGreen^+^ cells. On the left are the representative images. The arrowheads point to examples of tumor nodules. On the upper right are the quantifications of numbers of tumor nodules in lungs and the lung weights. *N* = 7. On the lower right are the representative images of H&E staining of the lungs. Scale bars are 100 μm. In all bar graphs, error bars represent the standard error of the mean. *P < 0.05. **P < 0.01. ***P < 0.001.

It has been reported that caspase-3 promotes migration and invasion of colorectal cancer cells [26]. To determine whether the elevated migration and invasion in anastatic cells are due to any residual executioner caspase activity in these cells, we assessed the effect of caspase inhibition on migration in 116-PTX-ZsGreen^+^, 116-PTX-ZsGreen^−^ and 116-control cells. Treatment with caspase inhibitor Z-DEVD did not have any influence on migration of these three populations (Supplementary Fig. S3). To figure out the molecular mechanism underlying the anastasis-induced enhancement in migration and metastasis, we performed RNA sequencing with 116-PTX-ZsGreen^+^, 116-PTX-ZsGreen^−^ and 116-control cells. Principal component analysis (PCA) showed that these three populations had distinct transcriptomes (Fig. 3A). We focused on genes whose expressions in these three populations were positively or negatively correlated with migration and metastasis capacity. Gene expression analysis identified 10 genes with the highest expression in 116-PTX-ZsGreen^+^ cells and the lowest expression in 116-control cells (Fig. 3B, Supplementary Dataset 1). We tested the mRNA level of these 10 genes in all the ZsGreen^+^, the ZsGreen^−^ and the control populations. Among them, *BIRC3*, *SLC2A3,* and *HK2* were commonly upregulated in all ZsGreen^+^ populations (Fig. 3C, Supplementary Fig. S4).

Among these three genes, *BIRC3* drew our interest as it encodes cellular IAP2 (cIAP2), a protein that belongs to the inhibitor of apoptosis (IAP) protein family and has been reported to regulate migration [27, 28]. We confirmed the upregulation of cIAP2 protein in all ZsGreen^+^ populations (Fig. 3D, Supplementary Fig. S5A). To investigate the role of cIAP2 in the anastasis-induced enhancement in migration and invasion, we knocked down *BIRC3* in all cell populations (Fig. 3E, Supplementary Fig. S5B). While reduced *BIRC3* expression suppressed in vitro migration and invasion of all cell populations, the suppression was more pronounced in the ZsGreen^+^ cells than in the ZsGreen^−^ and the control cells (Fig. 3F, G, Supplementary S5C, D). Consistently, overexpression of *BIRC3* in HCT-116 cells increased migration and invasion (Fig. 3H). We next injected the ZsGreen^+^ cells expressing sh*BIRC3* or shNC into nude mice through a tail vein to evaluate the effect of knocking down *BIRC3* on cancer cell colonization in the lungs. Cells with reduced *BIRC3* formed significantly fewer tumor nodules in the lungs (Fig. 3I, Supplementary Fig. S5E). These data together indicate that the enhanced migration and metastasis in the anastatic cells rely on increased expression of cIAP2.

### cIAP2 activates NFκB signaling to drive migration in the anastatic cells

**Fig. 4:**
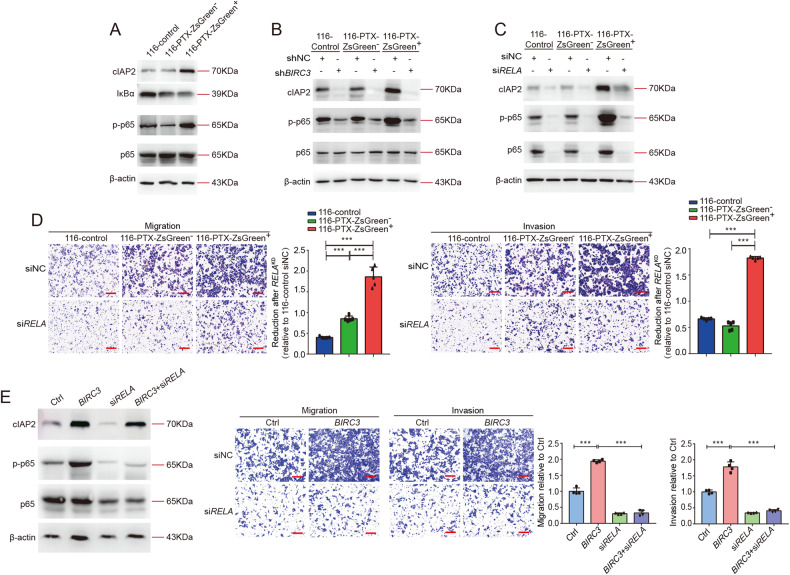
cIAP2 activates NF-κB signaling to drive migration. **A** Western blots showing the protein levels of cIAP2, IκBα, p-p65, and p65 in 116-control, 116-PTX-ZsGreen^−^ and 116-PTX-ZsGreen^+^ cells. **B**, **C** Western blots showing the effect of *BIRC3* knockdown (**B**) and *RELA* knockdown (**C**) on the protein levels of cIAP2, p-p65, and p65 in the indicated cells. **D** The effect of knocking down *RELA* on cell migration and invasion. Scale bars are 50 μm. The bar graphs show reduction after knocking down *RELA* (*RELA*^*KD*^). Data were normalized to 116-control siNC. *N* = 5. **E** The effect of knocking down *RELA* on migration and invasion of the *BIRC3*-overexpressing HCT-116 cells. On the Left are Western blots showing the protein levels of cIAP2, p-p65, and p65. Scale bars are 50 μm. *N* = 4. In all bar graphs, error bars represent the standard error of the mean. *P < 0.05. **P < 0.01. ***P < 0.001.

The next question is how the upregulated cIAP2 drives migration. cIAP2 has been reported as a positive regulator of NFκB signaling [29, 30]. Consistently, we detected increased p65 phosphorylation and reduced IκBα in all ZsGreen^+^ cells, indicating activation of NFκB signaling (Fig. 4A, Supplementary Fig. S6A). Knocking down *BIRC3* suppressed p65 phosphorylation in all cell populations especially in the ZsGreen^+^ cells (Fig. 4B, Supplementary Fig. S6B), suggesting cIAP2 is required for NFκB activation. Similar to what we observed with *BIRC3* knockdown, inhibition of NFκB signaling by chemical inhibitor QNZ or siRNA targeting *RELA*, the gene encoding p65, suppressed migration and invasion and attenuated the difference between different populations (Fig. 4C, D, Supplementary Figs. S7A, B, S8). However, we noticed that when NFκB was inhibited, either by chemical inhibitor or by siRNA, cIAP2 level was also reduced (Fig. 4C, Supplementary Figs. S7A, S8A, B). This is consistent with previous reports that *BIRC3* is a target gene of NFκB [31, 32]. To clarify the role of NFκB in cIAP2-triggered migration, we blocked NFκB signaling in cells overexpressing *BIRC3*. Inhibition of NFκB did not reduce cIAP2 protein level but suppressed the increased migration and invasion in *BIRC3*-overexpressing cells (Fig. 4E, Supplementary fig. S7C), indicating cIAP2 drives migration through activating NFκB signaling. The fact that cIAP2 expression and NFκB activity rely on each other suggests cIAP2 and NFκB signaling form a positive feedback loop to maintain high NFκB activity and high cIAP2 expression in the anastatic cells, making the anastatic cells more migratory.

### Anastatic cells acquire enhanced chemoresistance through upregulated cIAP2

**Fig. 5:**
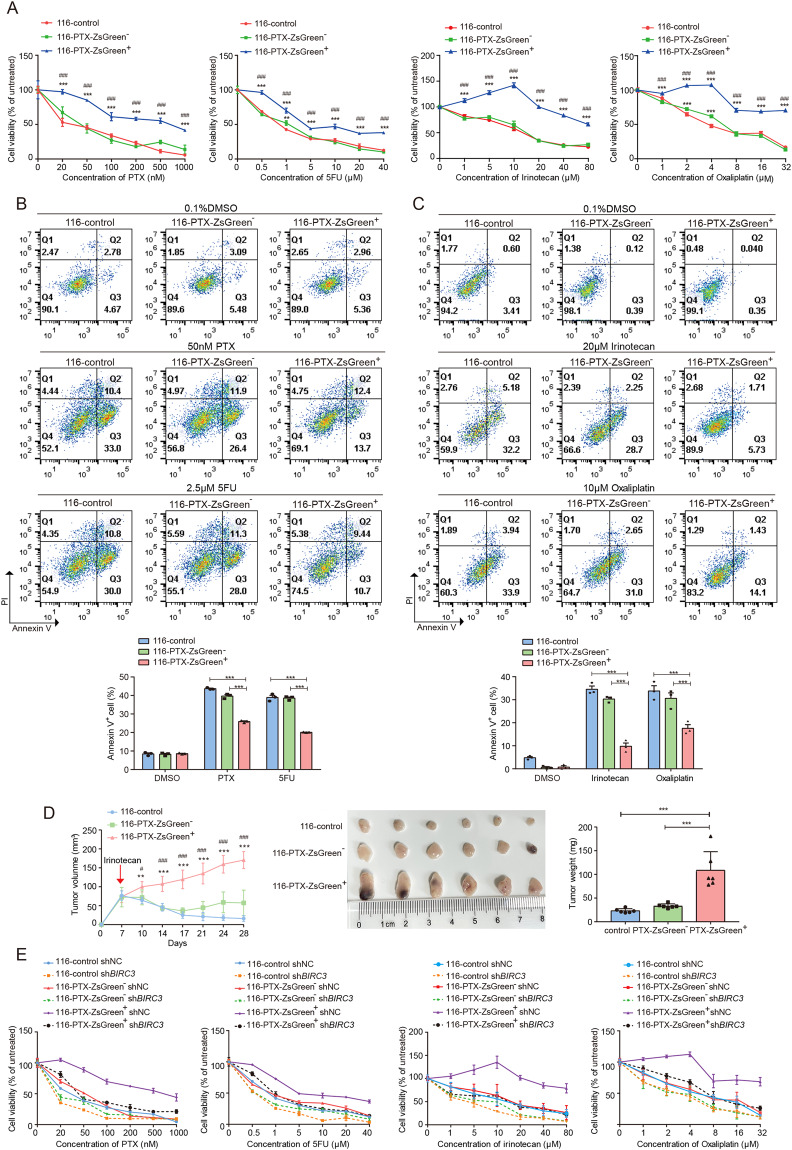
Anastatic cells acquire enhanced drug resistance through upregulated *BIRC3*. **A** The results of MTT assays showing the viability of 116-control, 116-PTX-ZsGreen^−^ and 116-PTX-ZsGreen^+^ cells after 48 h treatment with different concentrations of PTX, 5FU, irinotecan or oxaliplatin. *N* = 6. **B** Annexin V-PI staining to detect apoptosis in 116-control, 116-PTX-ZsGreen^−^ and 116-PTX-ZsGreen^+^ cells after 48 h treatment with 0.1% DMSO, 50 nM PTX or 2.5 μM 5FU. *N* = 3. **C** Annexin V-PI staining to detect apoptosis in 116-control, 116-PTX-ZsGreen^−^ and 116-PTX-ZsGreen^+^ cells after 48 h treatment with 0.1% DMSO, 20 μM irinotecan or 10 μM oxaliplatin. *N* = 3. **D** Growth of tumors formed by 116-control, 116-PTX-ZsGreen^−^ and 116-PTX-ZsGreen^+^ cells in nude mice with irinotecan treatment. On the left are the growth curves of tumors formed by the indicated cell populations. *N* = 6. The red arrow shows the time of first irinotecan injection. In the middle is the image of tumors collected after six times of irinotecan injection. On the right is the summary of tumor weights. *N* = 6. **E** The effect of *BIRC3* knockdown on cell viability upon PTX, 5FU, irinotecan, or oxaliplatin treatment. *N* = 6. Error bars represent the standard error of the mean. * or ^#^*P* < 0.05. ** or ^##^*P* < 0.01. *** or ^###^*P* < 0.001. **A**, **D** * represents significant difference compared to 116-control and ^#^ represents a significant difference compared to the ZsGreen^−^ group.

cIAP2 has been reported to promote chemoresistance in diverse types of cancer including colorectal cancer [33–36]. The upregulated cIAP2 expression in anastatic cells suggests they may be more resistant to chemotherapy than the ZsGreen^−^ cells and the control cells. To testify this speculation, we evaluated the sensitivity of 116-control, 116-PTX-ZsGreen^−^ and 116-PTX-ZsGreen^+^ cells to PTX. 116-PTX-ZsGreen^+^ cells exhibited higher resistance to PTX than 116-PTX-ZsGreen^−^ cells and 116-control whereas the latter two were similarly resistant (Fig. 5A, B). We also tested the sensitivity of these three groups of cells to other chemotherapeutic drugs like 5FU, oxaliplatin, and irinotecan. 116-PTX-ZsGreen^+^ cells showed stronger resistance to all these drugs compared to the other two groups of cells (Fig. 5A–C). Enhanced chemoresistance was also observed in 116–5FU-ZsGreen^+^ cells compared to 116-5FU-ZsGreen^−^ and 116-control cells (Supplementary Fig. S9A–C). We further evaluated chemoresistance to irinotecan in 116-PTX-ZsGreen^+^, 116-PTX-ZsGreen^−^ and 116-control cells in vivo. The three cell populations were inoculated subcutaneously into nude mice. When the xenografts were palpable, irinotecan was injected peritoneally twice a week. After six injections, the xenografts were harvested. Tumors formed by 116-PTX-ZsGreen^+^ cells were significantly larger than those formed by the other two groups (Fig. 5D), indicating anastatic cancer cells were more resistant to irinotecan in vivo. The difference in chemoresistance between different cell populations was dramatically attenuated by knocking down *BIRC3* (Fig. 5E, Supplementary Fig. S9D), indicating anastatic cells acquire elevated chemoresistance through upregulating cIAP2 expression.

### cIAP2/NFκB signaling is activated in response to chemotherapy to promote anastasis

**Fig. 6:**
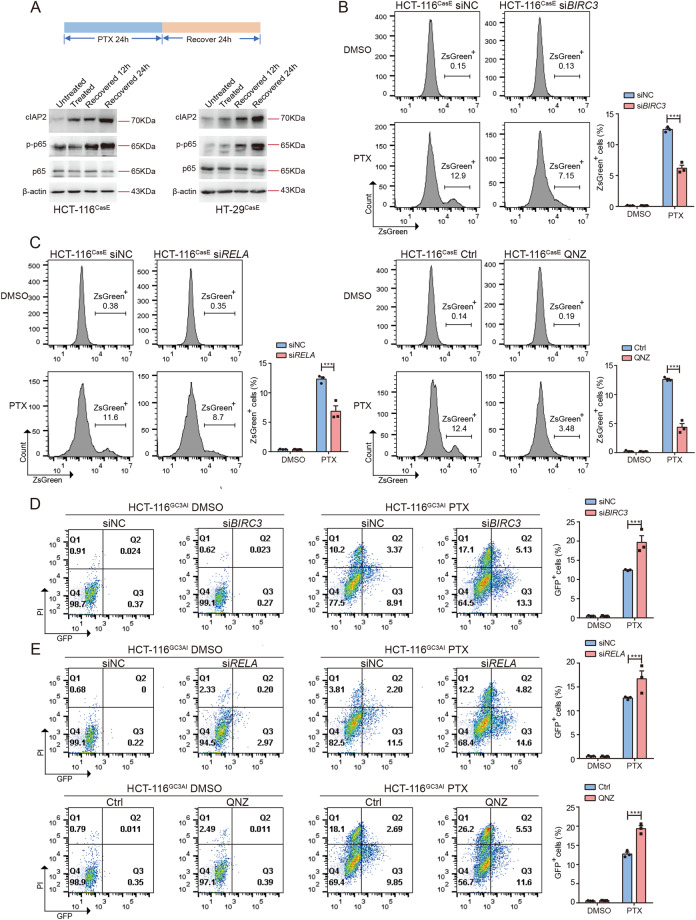
cIAP2/NFκB signaling is activated in response to chemotherapy to promote anastasis. **A** The protein levels of cIAP2, p-p65, and p65 in HCT-116^CasE^ or HT-29^CasE^ cells treated with PTX for 24 h (treated) and recovered for 12 h or 24 h. **B** The effect of knocking down *BIRC3* on the percentage of ZsGreen^+^ cells at 48 h recovery after PTX treatment. *N* = 3. **C** The effect of knocking down *RELA* (left) or chemical inhibition of NFκB signaling (right) on the percentage of ZsGreen^+^ cells at 48 h recovery after PTX treatment. *N* = 3. **D** The effect of knocking down *BIRC3* on the percentage of GFP^+^ HCT-116^GC3AI^ cells after 24 h PTX treatment. *N* = 3. **E** The effect of knocking down *RELA* (upper) or chemical inhibition of NFκB signaling (lower) on the percentage of GFP^+^ HCT-116^GC3AI^ cells after 24 h PTX treatment. *N* = 3. In all bar graphs, error bars represent the standard error of the mean. *P < 0.05. **P < 0.01. ***P < 0.001.

Both NFκB and cIAP2 can promote survival [31, 37, 38]. We monitored NFκB activity and cIAP2 expression in HCT-116 and HT-29 cells during PTX treatment and after removal of PTX. Phosphorylation of p65 and the level of cIAP2 protein was increased after 24 h PTX treatment and further upregulated at 12 h and 24 h after removal of PTX (Fig. 6A). Upregulation of cIAP2 protein and NFκB activity were also observed at the end of irinotecan or oxaliplatin treatment and 24 h after removal of the drugs (Supplementary Fig. S10A). We then wondered whether upregulated NFκB activity and cIAP2 expression are essential for cells to survive executioner caspase activation. Knocking down *BIRC3* or inhibition of NFκB during PTX treatment and 48 h recovery significantly reduced the percentage of ZsGreen^+^ cells (Fig. 6B, C, Supplementary Fig. S10B, C). To determine whether the reduced ZsGreen^+^ fraction is due to inhibited anastasis or reduced executioner caspase activation, we assessed the effect of *BIRC3* knockdown or NFκB inhibition on PTX-induced executioner caspase activation using a live executioner caspase activity reporter, GC3AI. GC3AI emits green fluorescence when cleaved by active executioner caspases [39]. Suppression of *BIRC3* or NFκB increased the percentage of cells with executioner caspase activation (GFP^+^) upon PTX treatment (Fig. 6D, E, Supplementary Fig. S10D, E), suggesting that the reduced ZsGreen^+^ fractions in *BIRC3* knockdown or NFκB-inhibited cells recovered from PTX treatment was due to inhibition of anastasis. These data indicate that cIAP2 and NFκB activity is required for anastasis. To determine whether upregulated *BIRC3* expression or activation of NFκB depends on caspase activation, we treated cells with caspase inhibitor or knocked down *CASP3* and *CASP7*. Neither inhibition of caspase activity nor reducing caspase level suppressed cIAP2 level and NFκB activity (Supplementary Fig. S11A, B). In addition, mitochondrial outer membrane permeabilization was also not involved in cIAP2 upregulation and NFκB activation upon drug treatment (Supplementary Fig. S11C, D), suggesting that induction of these pro-survival signals may not depend on activation of apoptosis pathway.

## Discussion

In this study, we demonstrate that exposure to chemotherapeutic drugs activates NFκB and upregulates cIAP2 expression in colorectal cancer cells to promote anastasis. The activated NFκB and cIAP2 then form a positive feedback loop in the anastatic cells to promote migration and metastasis.

Our work demonstrates that anastasis confers colorectal cancer cells more migratory. Elevated migration after survival from stress-induced executioner caspase activation has also been reported in cervical cancer cells [17], breast cancer cells [21], melanoma cells [20], and ovarian cancer cells [22], suggesting enhanced migration may be a common phenotypic change accompanying anastasis. Apoptosis and executioner caspases have been linked to cell migration and cancer metastasis. Apoptosis incidence and caspase-3 expression are positively correlated with lymph node metastasis in patients with squamous carcinoma of the tongue, gastric carcinoma, and ovarian cancer [40–42]. Caspase-3 is essential for migration in colorectal cancer cells, breast cancer cells, and lung cancer cells [26, 43]. However, in our study, the anastatic cells maintain enhanced migration after being separated from apoptotic cells and long-term culture. Inhibition of executioner caspases did not influence the migration of all cell populations. These data indicate that executioner caspase activity is not required for maintaining the enhanced migration in anastatic cells. This is consistent with the previous report on melanoma cells from Berthenet et al. [20].

Although several types of cancer cells have been known to acquire enhanced migration after anastasis, the underlying molecular mechanism varies. Previously, we found inhibition of TGFβ signaling partially suppressed anastasis-induced migration in HeLa cells after transient exposure to ethanol [17]. Seervi et al. reported that inhibition of nuclear export reversed the elevated migration in anastatic breast cancer cells [21]. Work by Berthenet et al. demonstrated that melanoma cells that survived executioner caspase activation became more motile due to hyperactivation of JNK [20]. In this study, we showed that the enhanced migration in anastatic colorectal cancer cells was due to upregulated cIAP2 and NFκB. cIAP2 belongs to IAP family. IAPs have both positive and negative effects on cell migration through different downstream molecules. XIAP and cIAPs suppress cell migration by promoting ubiquitination and degradation of c-Raf and Rac1 [44, 45]. On the other hand, XIAP can drive cancer cell migration by inhibiting RhoGDP dissociation inhibitor (RhoGDI) [46, 47] or activating NFκB [27]. cIAP2 promotes lymph node metastasis of gallbladder cancer by triggering NFκB activation [30] while driving colonic epithelial migration by increasing the abundance and activity of Rac1 [48]. In this study, we demonstrated that cIAP2 enhanced migration in anastatic colorectal cancer cells in an NFκB-dependent manner, supporting the role of cIAP2 as a positive regulator of migration. NFκB has been implicated in the progression of a wide range of human cancer. It can drive cancer cell migration and metastasis through downstream molecules like STAT3, MMPs, or crosstalk with other pathways [38, 49–51]. In addition to enhanced migration, we demonstrated cIAP2-dependent elevation of resistance to chemotherapeutic drugs in anastatic colorectal cancer cells. cIAP2 has been linked to drug resistance in pancreatic cancer, colorectal cancer, and oral squamous cell carcinoma [33, 35, 52]. Our work supports the role of cIAP2 in chemoresistance.

We further showed reducing cIAP2 expression or NFκB activity suppressed anastasis in colorectal cancer cells exposed to chemotherapeutic drugs. Recently, we reported that when exposed to apoptotic stimuli, ovarian cancer cells with relatively lower executioner caspase activity had higher chance to survive than those with high executioner caspase activity [22]. Valon et al. also demonstrated that in Drosophila notum epithelium, cells in which amplification of executioner caspase activation was blocked survived [53]. This work suggests that whether stressed cells can undergo anastasis may depend on the dynamics of executioner caspase activation. cIAP2 can inhibit caspase-3 and 7 [37, 54]. Its upregulation in cells with executioner caspase activation initiated may help prevent rapid amplification of executioner caspase activity, and therefore, promotes anastasis. The molecular mechanism underlying the regulation of anastasis by cIAP2, NFκB, and other regulators identified from previous studies needs more effort to elucidate in the future.

In summary, we report that colorectal cancer cells can survive through anastasis upon exposure to chemotherapeutic drugs via upregulation of cIAP2 and activation of NFκB. The positive feedback loop between cIAP2 and NFκB sustains a high level of cIAP2 expression and NFκB activity in anastatic cells to confer them more migratory. Our study unveils cIAP2/NFκB and anastasis as important regulators for post-chemotherapy metastasis.

## Supplementary information

Supplementary figures and tables

Supplementary dataset 1

Original Western blots

reproducibility checklist

## Data Availability

The raw data for RNA sequencing can be assessed at NCBI with accession number PRJNA906751. All the other raw data supporting the findings of this study are available from the corresponding authors upon request.
