# Supplementary material for: Anastasis enhances metastasis and chemoresistance of colorectal cancer cells through upregulating cIAP2/NFκB signaling
Source: Cell Death Dis. 2023 Jun 30;14(6):388. doi: 10.1038/s41419-023-05916-8 (PMC10313691; doi:10.1038/s41419-023-05916-8)
Supplement: Supplementary file 1 — Supplementary figures and tables [file 41419_2023_5916_MOESM1_ESM.pdf]

Supplementary information

Anastasis enhances metastasis and chemoresistance of colorectal cancer cells through upregulating cIAP2/NFκB signaling

Wang et al.

This file contains the following:

Supplementary figure S1-S11 and their legends

Supplementary table S1-S3 and their legends

Legend for Supplementary dataset 1

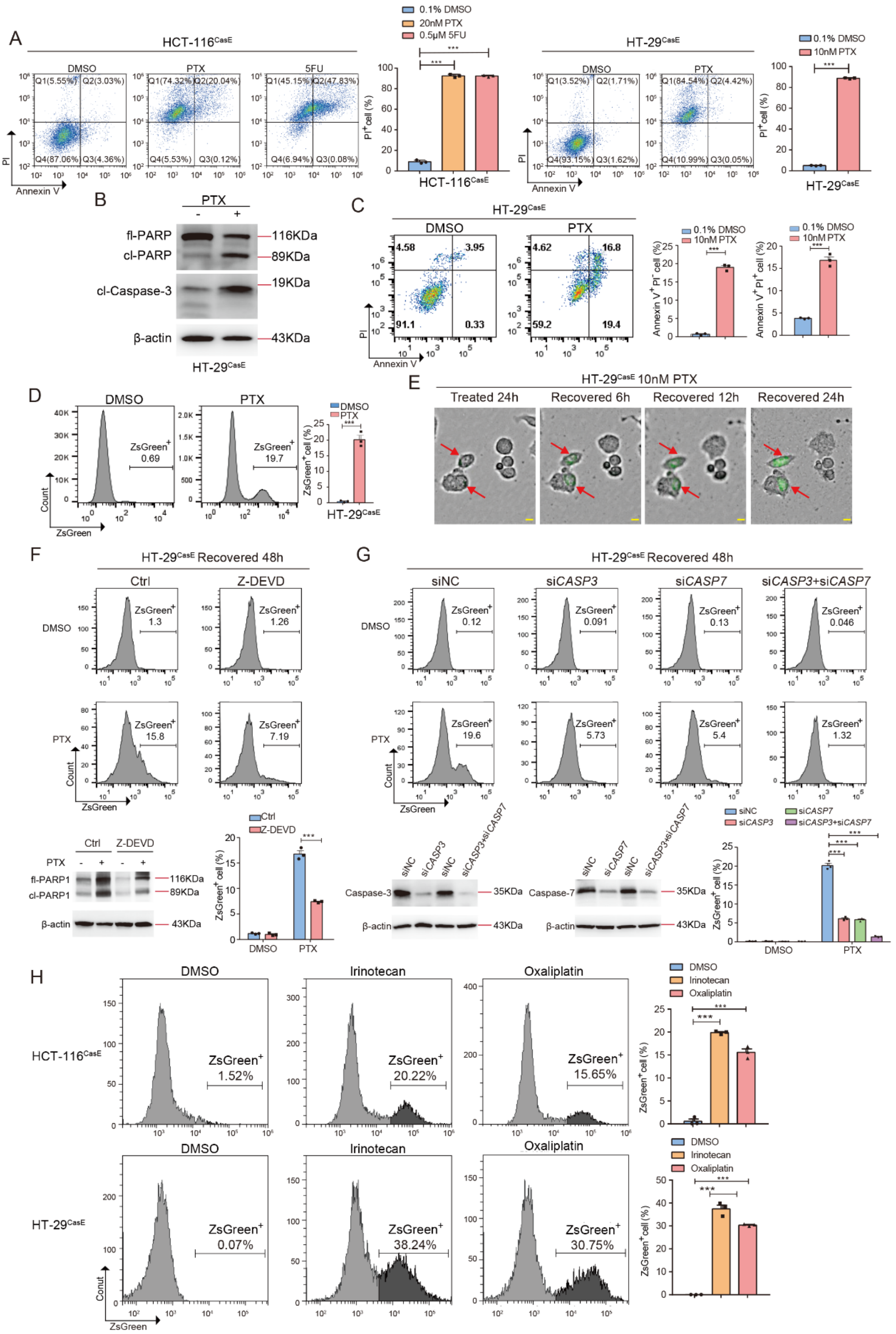

Supplementary figure S1. Anastasis occurs in colorectal cancer cells after treatment with chemotherapeutic drugs.

A) Annexin V-PI staining to detect cell death after 96 h of PTX or 5FU treatment on HCT-116^{CasE} or HT-29^{CasE} cells. N = 3. B) Western blots of full-length PARP1 (fl-PARP1), cleaved PARP1 (cl-PARP1) and cleaved caspase-3 (cl-caspase-3) in HT-29^{CasE} cells after 24 h treatment with 10 nM PTX or 0.1% DMSO (-). C) Annexin V-PI staining to detect apoptosis in HT-29^{CasE} cells after 24 h treatment with 10 nM PTX or 0.1% DMSO. N = 3. D) Quantification of the percentage of ZsGreen^{+} cells in HT-29^{CasE} cells after 48 h recovery from PTX treatment. N = 3. E) Representative images from time-lapse live imaging of HT-29^{CasE} cells during recovery after 24 h PTX treatment. The scale bars are 50 μm. The red arrows point to the cells undergoing anastasis. F & G) Analysis of the effect of caspase inhibitor Z-DEVD-fmk (Z-DEVD) (F) or siRNA targeting *CASP3* or/and *CASP7* (G) on the percentage of ZsGreen^{+} cells at 48 h recovery after PTX treatment. N = 3. The Western blots show the efficiency of executioner caspase inhibition (F) or knockdown by siRNA (G). H) Quantification of the percentage of ZsGreen^{+} cells in HCT-116^{CasE} or HT-29^{CasE} cells after 48 h recovery from irinotecan or oxaliplatin treatment. N = 3. In all bar graphs, error bars represent the standard error of the mean. *: $P < 0.05$. **: $P < 0.01$. ***: $P < 0.001$.

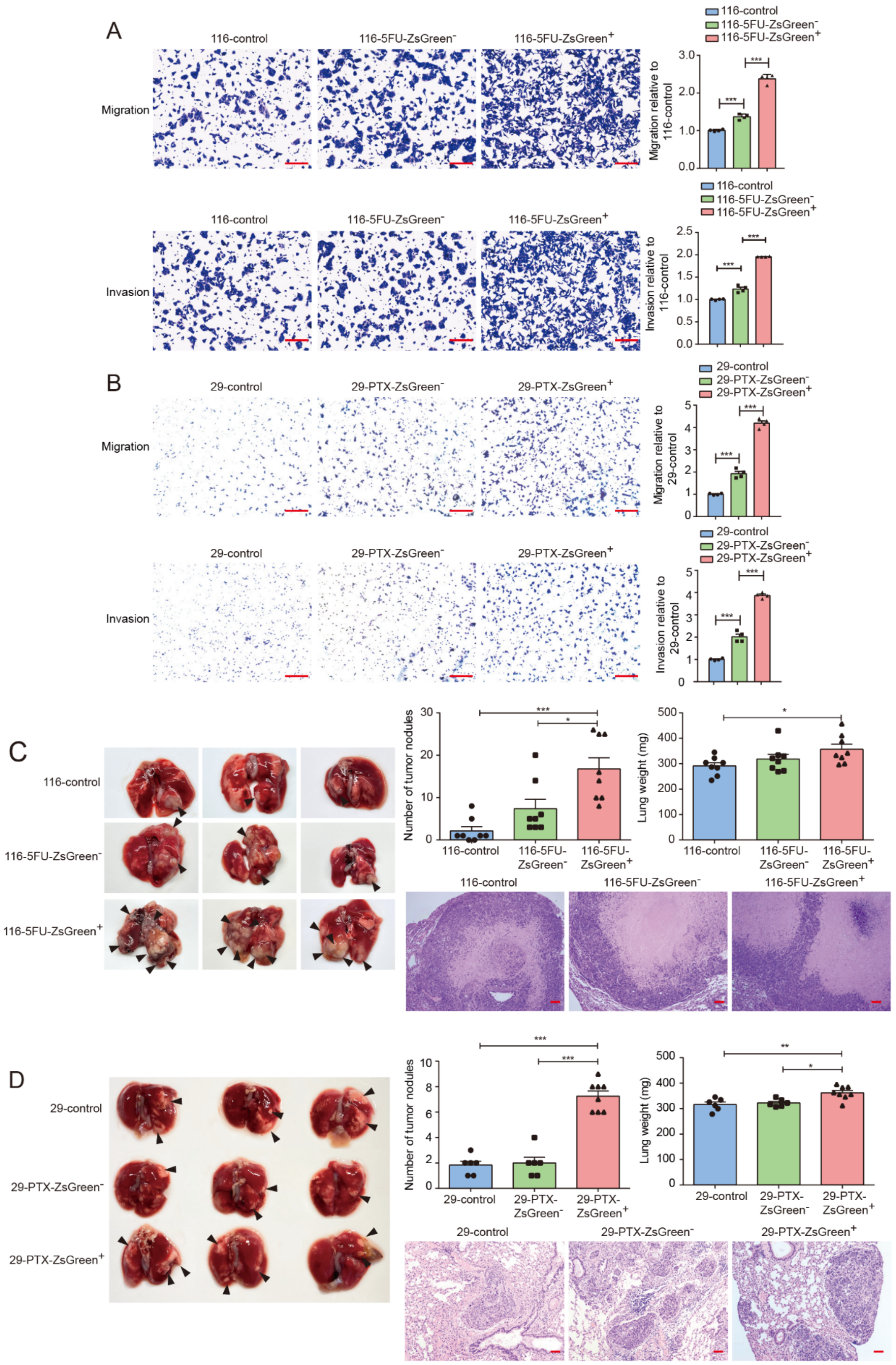

Supplementary figure S2. Anastatic colorectal cancer cells acquire enhanced migration and metastasis.

A & B) Transwell assays to evaluate the migration and invasion capacity in the indicated cell populations. Scale bars are 50 μm. N = 4. C & D) Lung colonization of the indicated populations. The arrowheads point to examples of tumor nodules. N = 8 in 116-control, 116-5FU-ZsGreen$^-$ and 116-5FU-ZsGreen$^+$ groups. N = 6 in 29-control group and 29-PTX-ZsGreen$^-$ group and N = 8 in 29-PTX-ZsGreen$^+$ group. Scale bars in images of H & E staining are 100 μm. In all bar graphs, error bars represent the standard error of the mean. *: $P < 0.05$. **: $P < 0.01$. ***: $P < 0.001$.

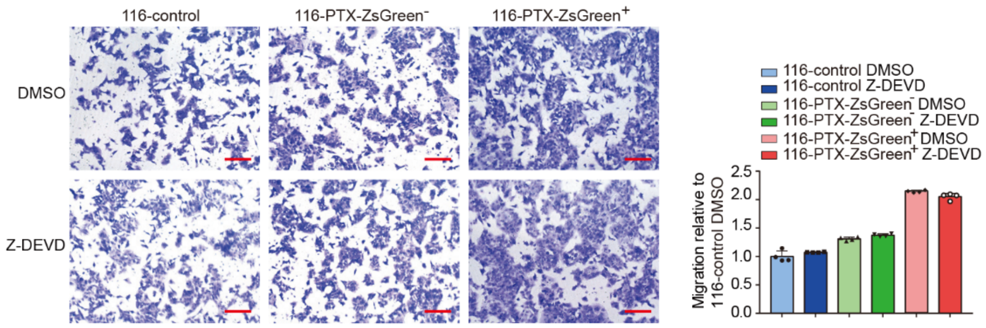

Supplementary figure S3. Inhibition of caspases does not affect migration.

Transwell migration assays of the indicated groups after 24 h treatment with 0.1% DMSO or 10 μM Z-DEVD. Scale bars are 50 μm. N = 4. Error bars represent the standard error of the mean.

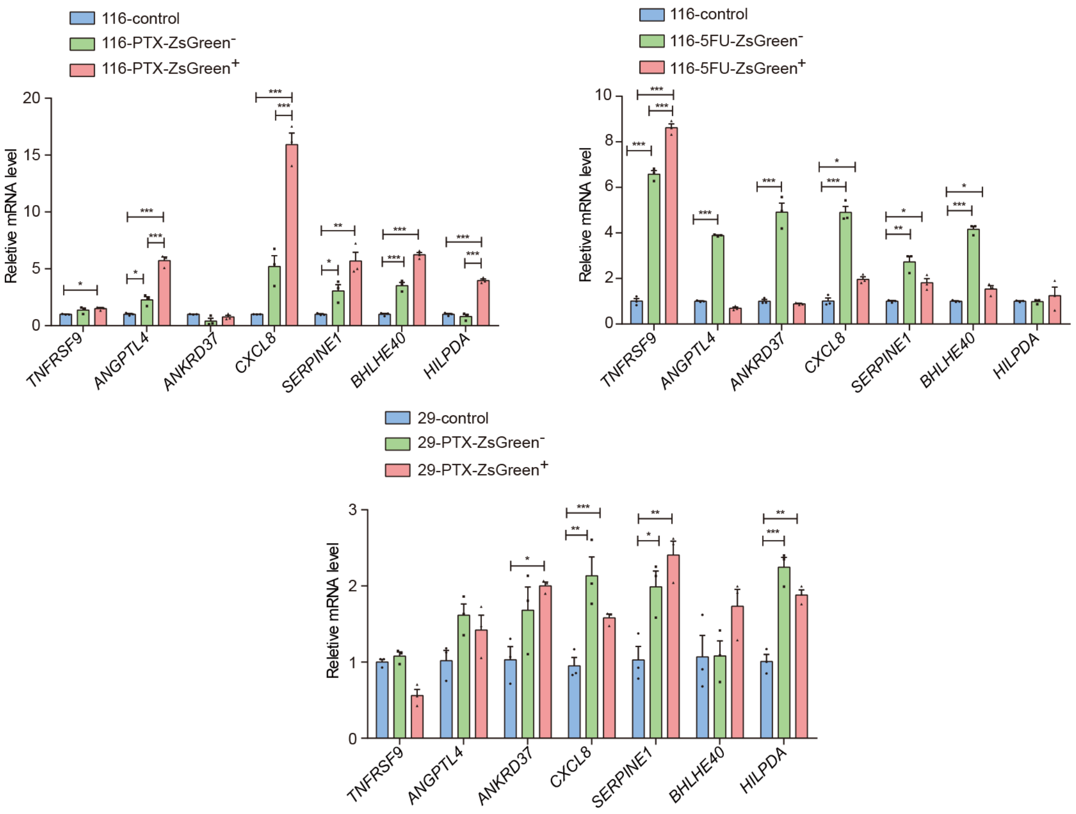

Supplementary figure S4. The mRNA expression of the upregulated genes identified from RNAseq.

The mRNA expression of *TNFRSF9*, *ANGPTL4*, *ANKRD37*, *CXCL8*, *SERPINE1*, *BHLHE40*, *HILPDA* in the indicated cell populations. N = 3. Error bars represent the standard error of the mean. *: $P < 0.05$. **: $P < 0.01$. ***: $P < 0.001$.

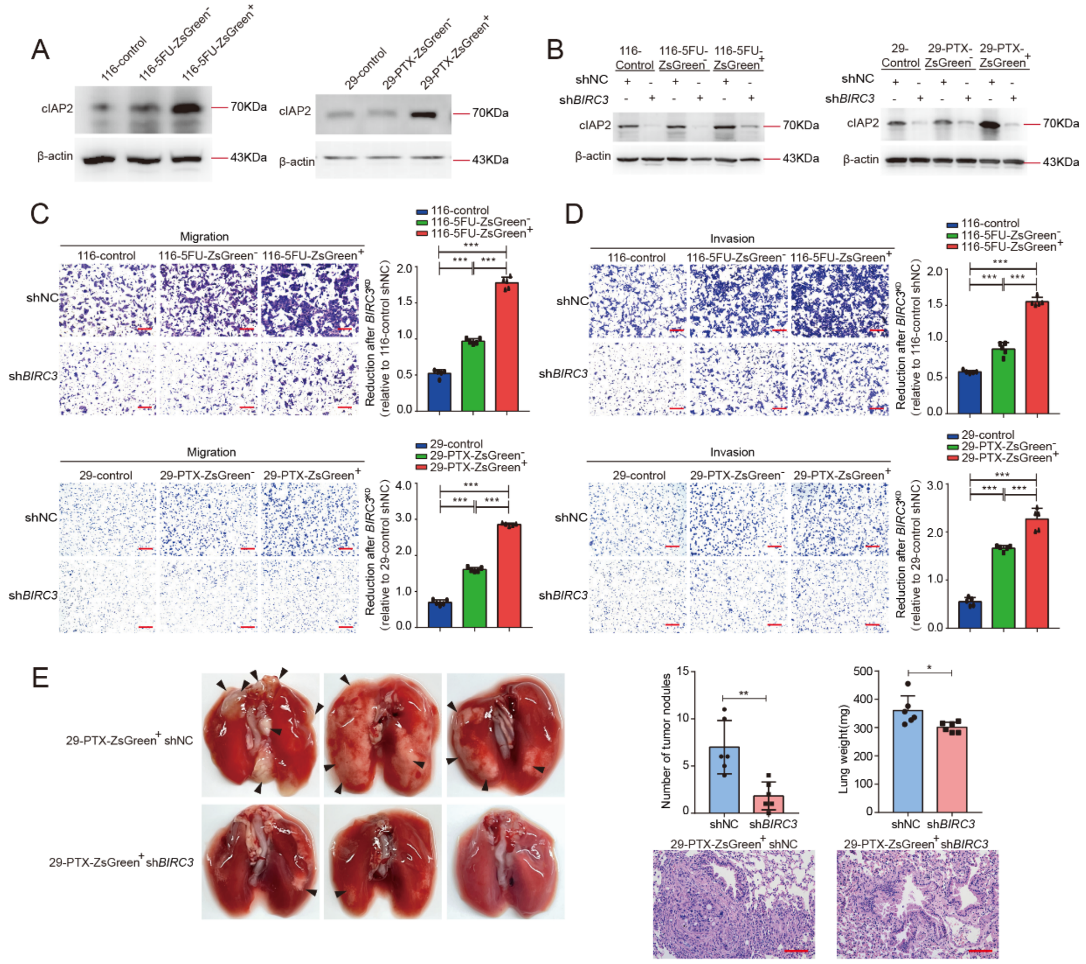

Supplementary figure S5. The enhanced migration and metastasis in anastatic cells are mediated by upregulation of *BIRC3*.

A & B) The protein levels of cIAP2 in the indicated populations. C & D) The effect of *BIRC3* knockdown on cell migration (C) and invasion (D). Scale bars are 50 μm. The bar graphs show reduction after knocking down *BIRC3* (*BIRC3*^{KD}). Data were normalized to 116-control shNC or 29-control shNC. N = 5. E) The effect of knocking down *BIRC3* on lung metastasis of 29-PTX-ZsGreen^{+} cells. On the left are the representative images. The arrowheads point to examples of tumor nodules. On the upper right are the quantifications of numbers of tumor nodules in lungs and the lung weights. N = 6. On the lower right are the representative images of H & E staining of

the lungs. Scale bars are 100 μm. In all bar graphs, error bars represent the standard error of the mean. *: $P < 0.05$. **: $P < 0.01$. ***: $P < 0.001$.

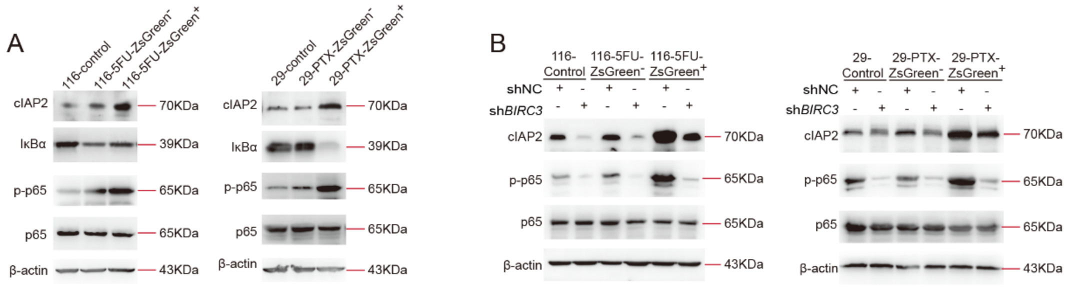

Supplementary figure S6. Activation of NFκB in anastatic cells relies on upregulated *BIRC3*.

Western blots showing the protein levels of cIAP2, IκBα, p-p65 and p65 in the indicated cell populations.

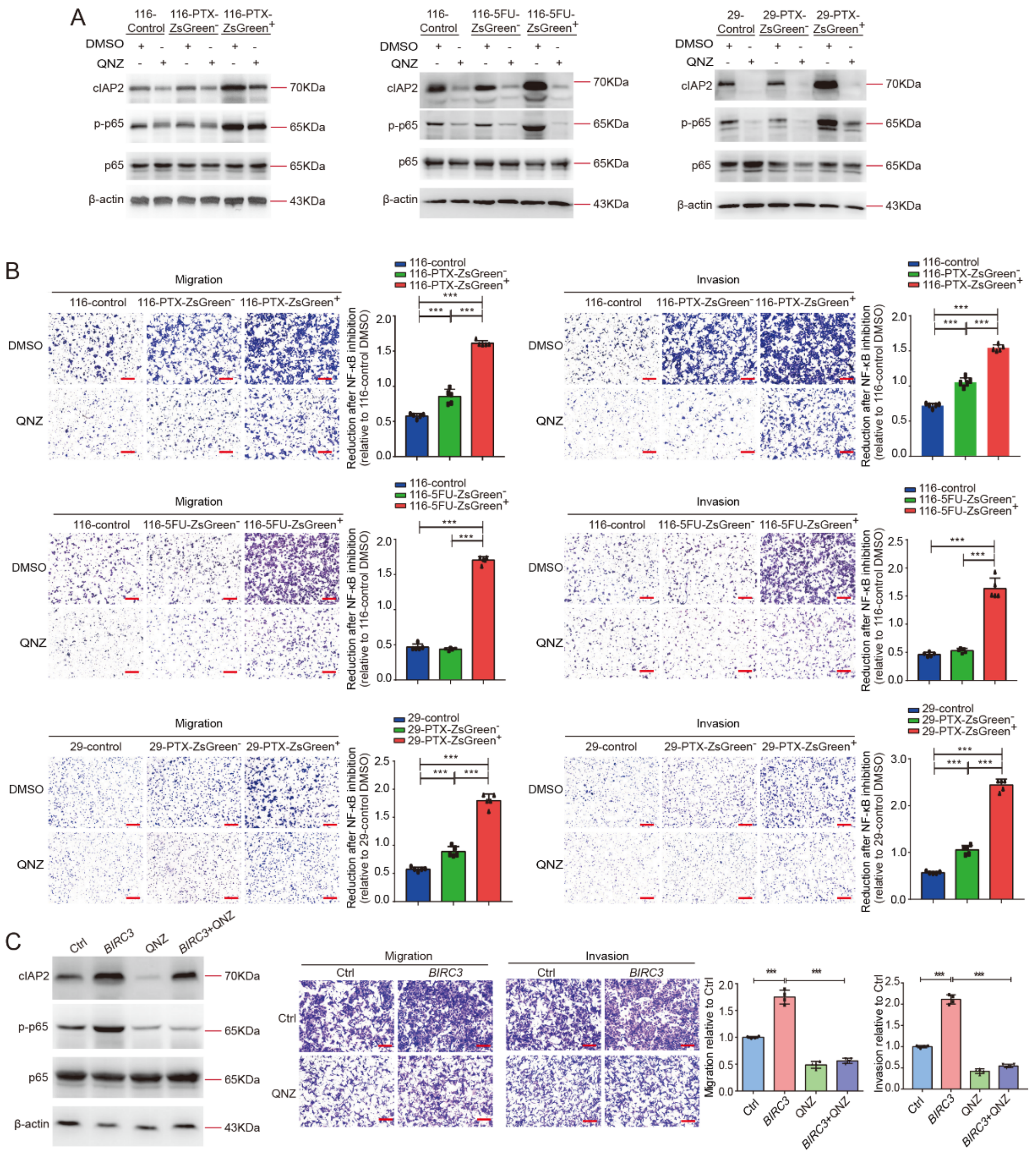

Supplementary figure S7. Inhibition of NFκB activity suppresses enhanced migration and invasion in anastatic cells and *BIRC3*-overexpressing cells.

A) Western blots showing the protein levels of cIAP2, p-p65 and p65 in the indicated cell populations. B) The effect of inhibition of NFκB signaling by QNZ on cell migration and invasion. N = 5. Scale bars are 50 µm. The bar graphs show reduction after NFκB inhibition. Data were normalized to 116-control DMSO or 29-control DMSO. C) The effect of NFκB inhibition on migration and invasion of HCT-116 cells overexpressing *BIRC3*. On the Left are Western blots showing the protein levels of

cIAP2, p-p65 and p65. Scale bars are 50 μm. N = 4. In all bar graphs, error bars represent the standard error of the mean. *: $P < 0.05$. **: $P < 0.01$. ***: $P < 0.001$.

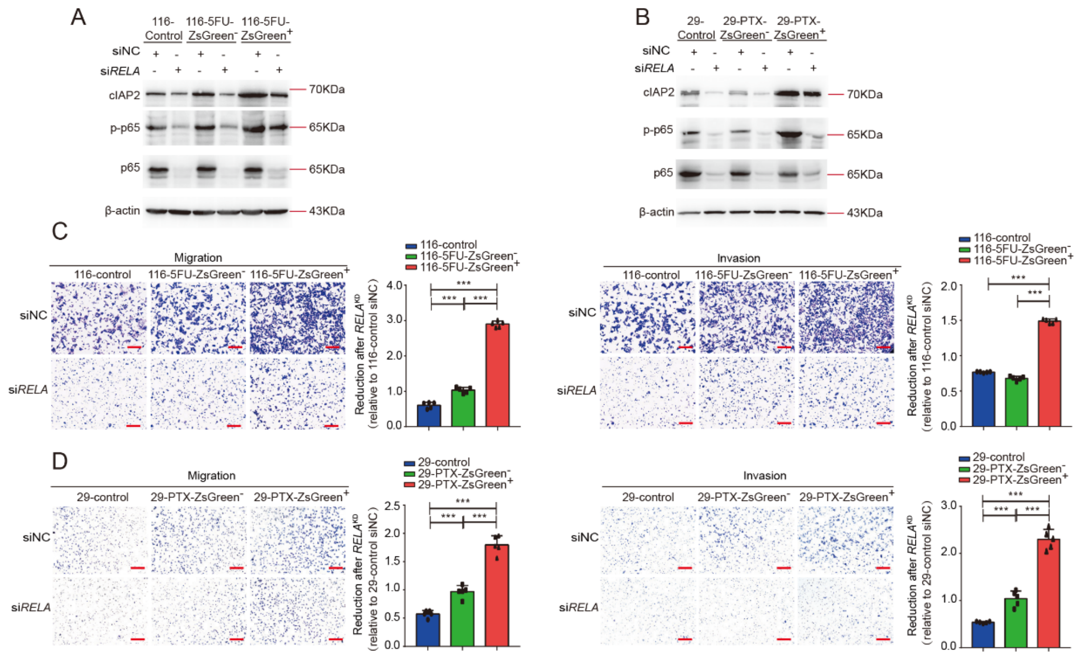

Supplementary figure S8. Knocking down *RELA* suppresses enhanced migration and invasion in anastatic cells.

A & B) Western blots showing the protein levels of cIAP2, p-p65 and p65 in the indicated cell populations. C & D) The effect of knocking down *RELA* on cell migration and invasion. Scale bars are 50 μm. The bar graphs show reduction after knocking down *RELA* ($RELA^{KD}$). Data were normalized to 116-control siNC (C) or 29-control siNC (D). N = 5. In all bar graphs, error bars represent the standard error of the mean. *: $P < 0.05$. **: $P < 0.01$. ***: $P < 0.001$.

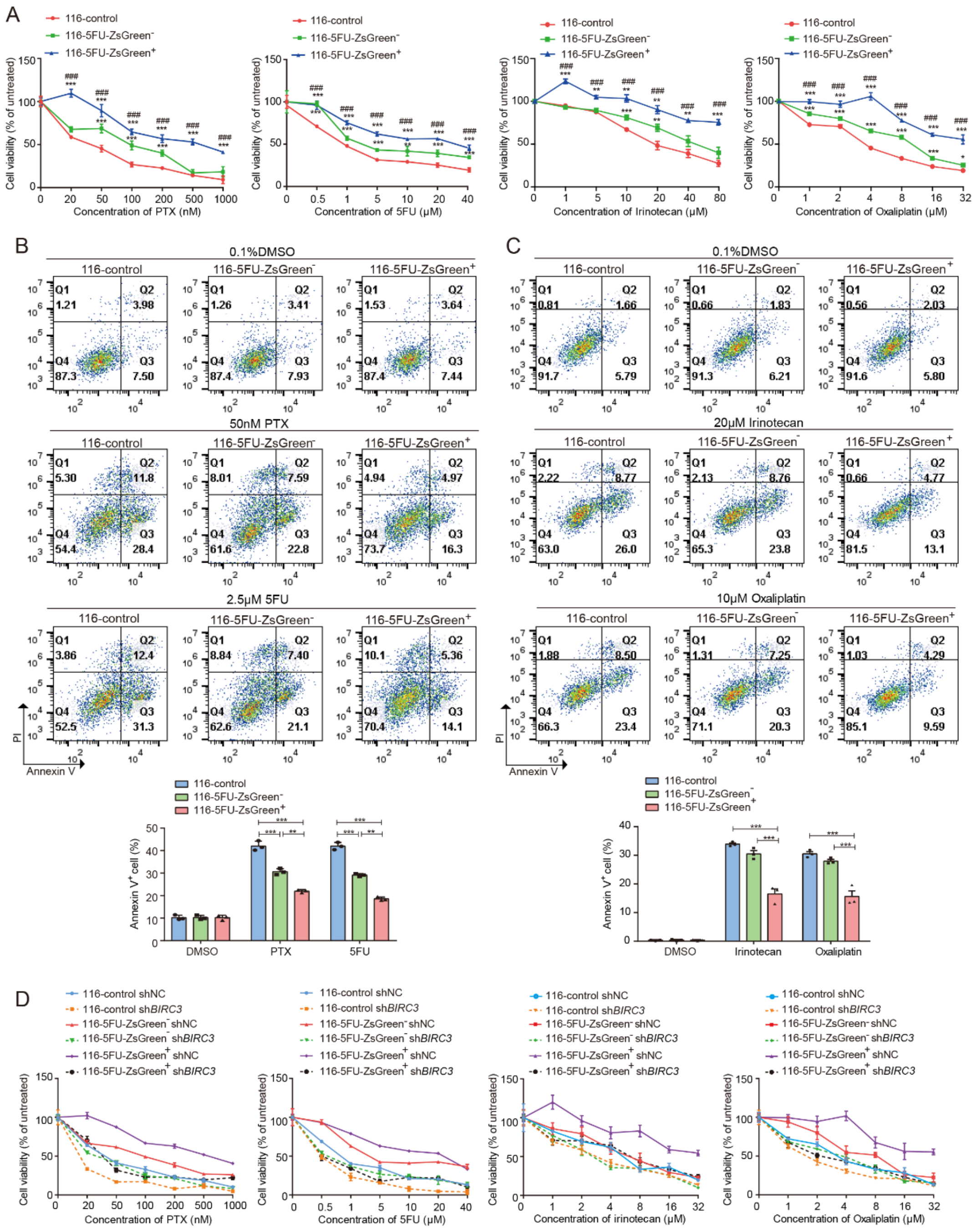

Supplementary figure S9. Anastatic cells gain enhanced drug resistance through upregulated *BIRC3*.

A) The results of MTT assays showing the viability of 116-control, 116-5FU-ZsGreen⁻ and 116-5FU-ZsGreen⁺ cells after 48 h treatment with different concentrations of PTX, 5FU, irinotecan or oxaliplatin. N = 6. * represents significant difference compared to 116-control and # represents significant difference compared to the ZsGreen⁻ group. B)

Annexin V-PI staining to detect apoptosis in 116-control, 116-5FU-ZsGreen$^-$ and 116-5FU-ZsGreen$^+$ cells after 48 h treatment with 0.1% DMSO, 50 nM PTX or 2.5 μM 5FU. N = 3. C) Annexin V-PI staining to detect apoptosis in 116-control, 116-5FU-ZsGreen$^-$ and 116-5FU-ZsGreen$^+$ cells after 48 h treatment with 0.1% DMSO, 20 μM irinotecan or 10 μM oxaliplatin. N = 3. D) The effect of *BIRC3* knockdown on cell viability upon PTX, 5FU, irinotecan or oxaliplatin treatment. N = 6. Error bars represent the standard error of the mean. * or #: $P < 0.05$. ** or ##: $P < 0.01$. *** or ###: $P < 0.001$.

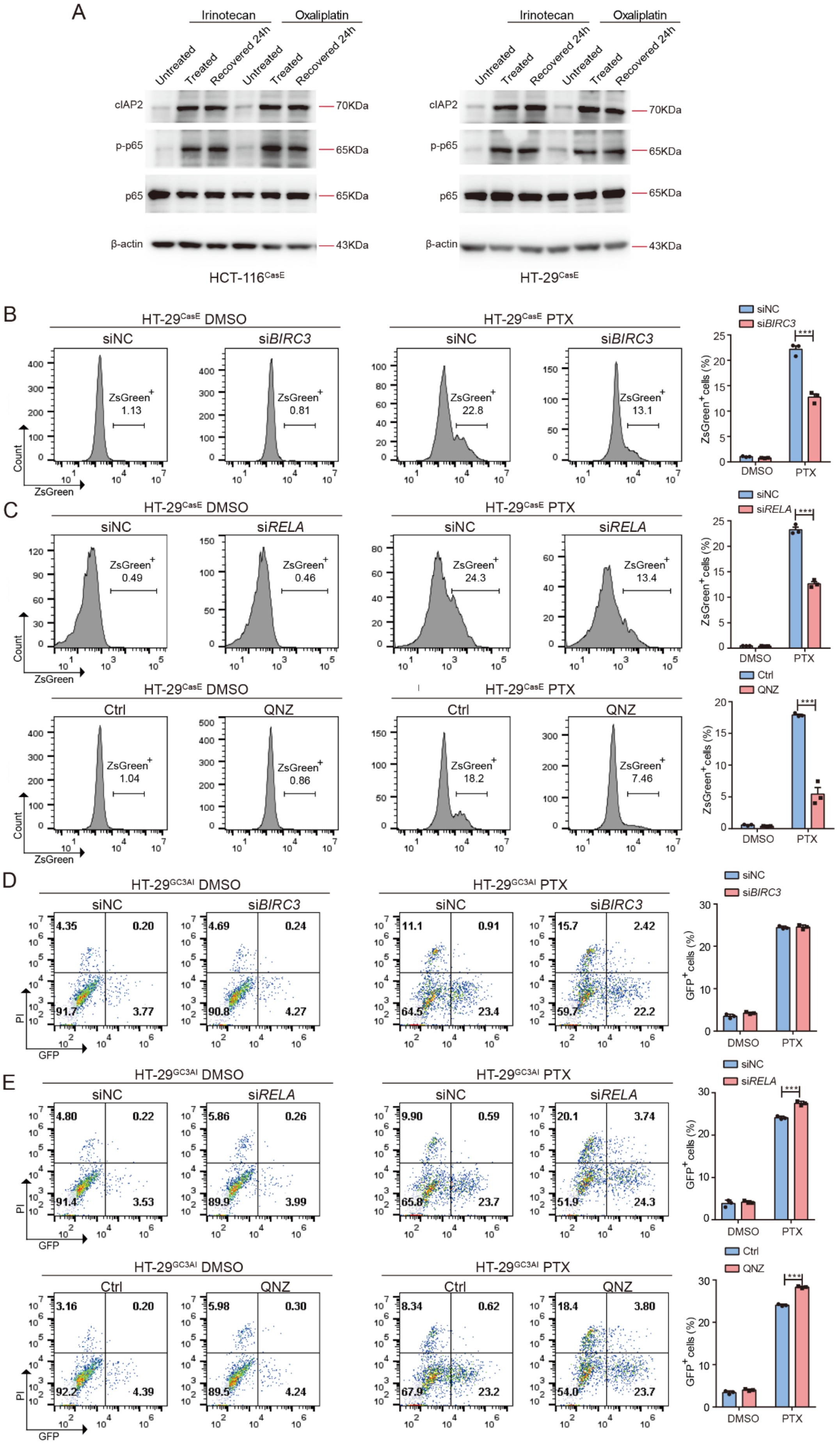
A
Irinotecan
Oxaliplatin
Untreated
Treated
Recovered 24h
cIAP2
p-p65
p65
β-actin
70KDa
65KDa
43KDa
HCT-116CasE
HT-29CasE
B
HT-29CasE DMSO
HT-29CasE PTX
siNC
siBIRC3
ZsGreen+
1.13
0.81
22.8
13.1
Count
ZsGreen
ZsGreen+ cells (%)
DMSO
PTX
C
siRELA
0.49
0.46
24.3
13.4
Ctrl
QNZ
1.04
0.86
18.2
7.46
D
HT-29GC3AI DMSO
HT-29GC3AI PTX
PI
GFP
GFP+ cells (%)
E

Supplementary figure S10. cIAP2/NFκB signaling is activated in response to chemotherapy to promote anastasis.

A) The protein levels of cIAP2, p-p65 and p65 in HCT-116^{CasE} or HT-29^{CasE} cells treated with irinotecan or oxaliplatin for 24 h (treated) and recovered for 24 h. B) The effect of knocking down *BIRC3* on the percentage of ZsGreen^{+} cells in HT-29^{CasE} cells at 48 h recovery after PTX treatment. N = 3. C) The effect of knocking down *RELA* or chemical inhibition of NFκB signaling on the percentage of ZsGreen^{+} cells in HT-29^{CasE} cells at 48 h recovery after PTX treatment. N = 3. D) The effect of knocking down *BIRC3* on the percentage of GFP^{+} HT-29^{GC3AI} cells after 24 h PTX treatment. N = 3. E) The effect of knocking down *RELA* (upper) or chemical inhibition of NFκB signaling (lower) on the percentage of GFP^{+} HT-29^{GC3AI} cells after 24 h PTX treatment. N = 3. In all bar graphs, error bars represent the standard error of the mean. *: $P < 0.05$. **: $P < 0.01$. ***: $P < 0.001$.

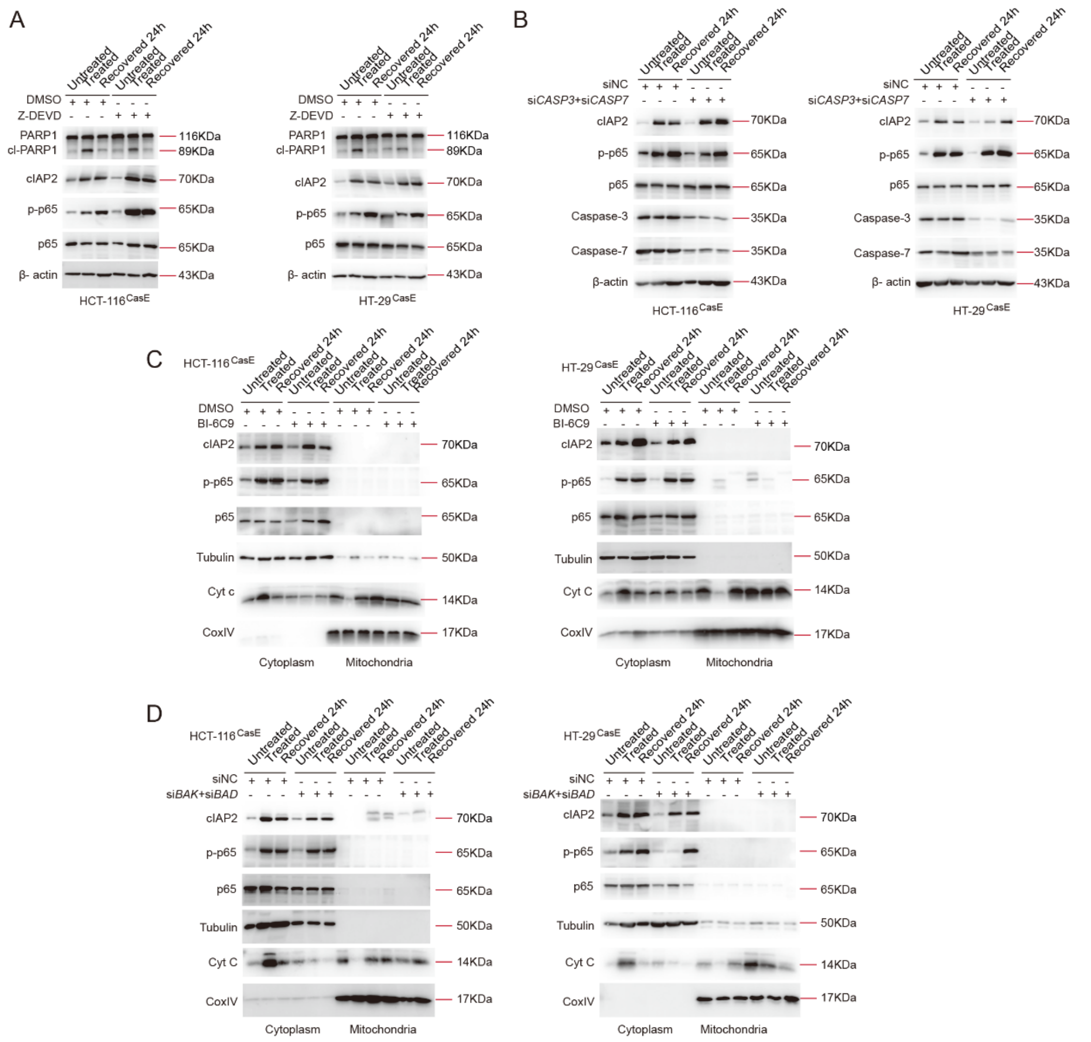

Supplementary figure S11. Upregulation of cIAP2 and activation of NFκB upon treatment with chemotherapeutic drugs do not rely on activation of caspases and MOMP.

A) The effect of caspase inhibitor DEVD on cIAP2 level and p65 phosphorylation after PTX treatment and after 24h recovery. B) The effect of knocking down both *CASP3* and *CASP7* on cIAP2 level and p65 phosphorylation after PTX treatment and after 24h recovery. C) The effect of treatment with Bid inhibitor BI-6C9, which can inhibit MOMP, on cIAP2 level and p65 phosphorylation after PTX treatment and after 24h recovery. D) The effect of knocking down *BAK* and *BAD* on cIAP2 level and p65

phosphorylation after PTX treatment and after 24h recovery. In (C) and (D), levels of cytochrome c (Cyt C) in cytosol and in mitochondria were used to monitor MOMP. COXIV is marker for mitochondria and tubulin is marker for cytosol fraction.

Supplementary table S1. The list of primers.

Primer name	Primer sequence (5'-3')
BIRC3 F	CTGTGATGGTGGACTCAGGTGT
BIRC3 R	ACTGGCTTGAACTTGACGGATG
HK2 F	ACAAGATAAAGGAAGTCACCAAAAT
HK2 R	TAAAAAAAAGAAAAACACAAAAACA
SLC2A3 F	GTATTTATTTTTATGCTCCTTCTGCTT
SLC2A3 R	ATTATCTCCCTCCTCTCTTTATTCTTT
TNFRSF9 F	TGTGCTTGTGAATGGGACGAA
TNFRSF9 R	AGAAACGGAGCGTGAGGAAG
ANGPTL4 F	GACGGTGACTCTTGGCTCTGC
ANGPTL4 R	GCTTCTCCAGTCGTGGTCTTCTT
ANKRD37 F	CCTGCTTGTAGCCAGTGATGC
ANKRD37 R	AAACTTGGCACAGTCTGGAAAT
CXCL8 F	ACATACTCCAAACCTTTCCACCC
CXCL8 R	GCCCTCTTCAAAAACTTCTCCAC
SERPINE1 F	GAGCAGGACGAACCGCC
SERPINE1 R	GAAACACCCTCACCCCGAAG
BHLHE40 F	AGTGGCTATGGAGGAGAATCGG
BHLHE40 R	AGGGCAGGCAGAAAGGAGG
HILPDA F	TGGATTGCTTATGGCTATGAGAT
HILPDA R	ACAGATGTTTAGGAAGTAGGGTTGA
Actin F	TGACGGGGTCACCCACACTGTGCCCATCTA
Actin R	CTAGAAGCATTTGCGGTGGACGATGGAGGG

Supplementary table S2. List of antibodies.

Antibodies	Source	Cat. No
anti-BIRC3	Cell Signaling Technology	Cat # 3031T
anti-p65	Cell Signaling Technology	Cat # 8482T
anti-p-p65	Cell Signaling Technology	Cat # 3033T
anti-IκBα	Cell Signaling Technology	Cat # 4814T
anti-PARP1	Cell Signaling Technology	Cat # 9532T
anti-cleaved Caspase-3	Cell Signaling Technology	Cat # 9661T
anti-Caspase-3	Cell Signaling Technology	Cat # 9662T
anti-Caspase-7	Cell Signaling Technology	Cat # 9492T
anti-β-actin	Santa Cruz Biotechnology	Cat # A2228
anti-Cytochrome c	Cell Signaling Technology	Cat # 4280T
anti- COX IV	Cell Signaling Technology	Cat # 4850T
anti-β3-Tubulin	Cell Signaling Technology	Cat # 5568T

Supplementary table S3. List of siRNA.

siRNA	Targeting sequence (5'-3')
siNC	UUCUCCGAACGUGUCACGUTT
si*RELA*	GATTGAGGAGAAACGTAAA
si*CASP3*	CCGACAAGCUUGAAUUUAUTT
si*CASP7*	GCCCAUCAAUGACACAGAUTT
si*BAK*	TCCCAACATCAACCGACGCTAT
si*BAD*	GGAGGAUGAGUGACGAUUTT

Supplementary dataset 1. List of differentially expressed genes from RNAseq.
