# Supplementary figures and images for: Anastasis enhances metastasis and chemoresistance of colorectal cancer cells through upregulating cIAP2/NFκB signaling
Source: Cell Death Dis. 2023 Jun 30;14(6):388. doi: 10.1038/s41419-023-05916-8 (PMC10313691; doi:10.1038/s41419-023-05916-8)

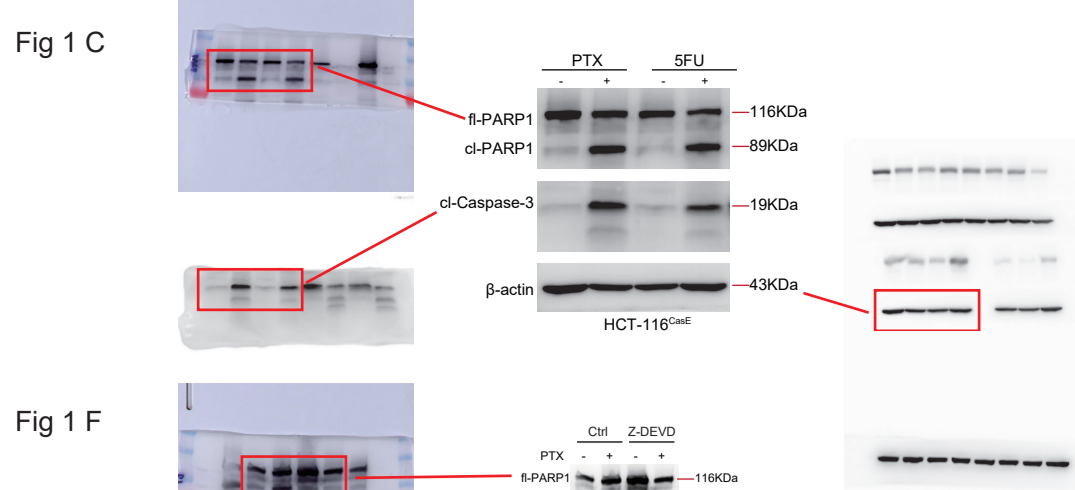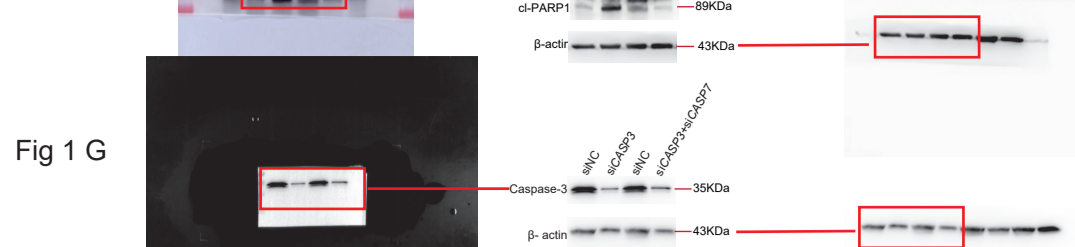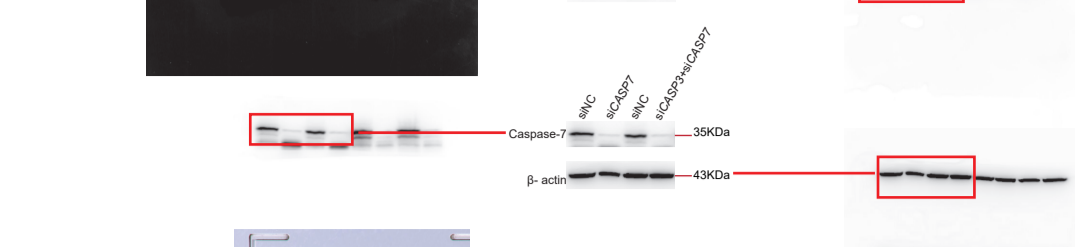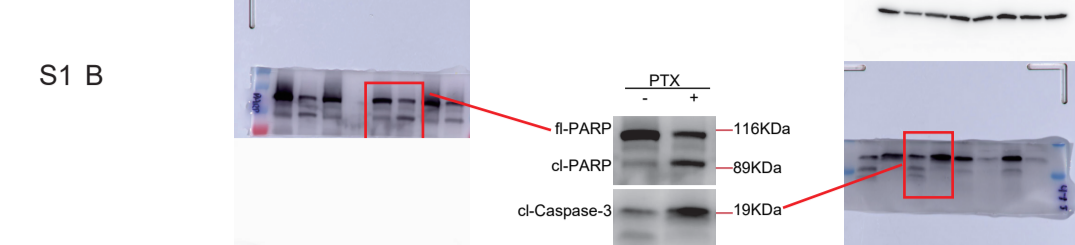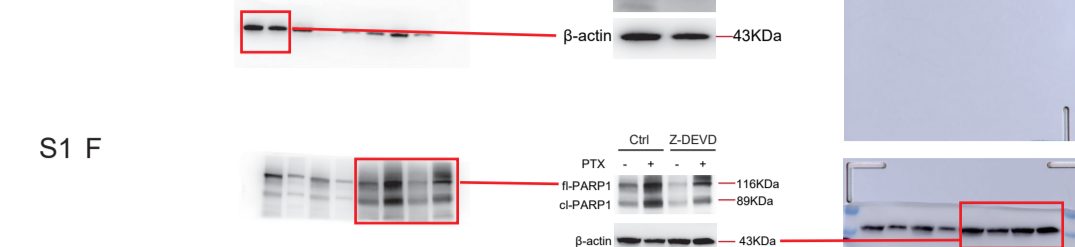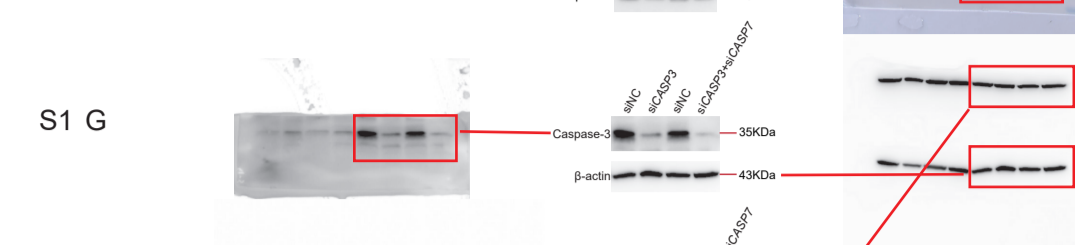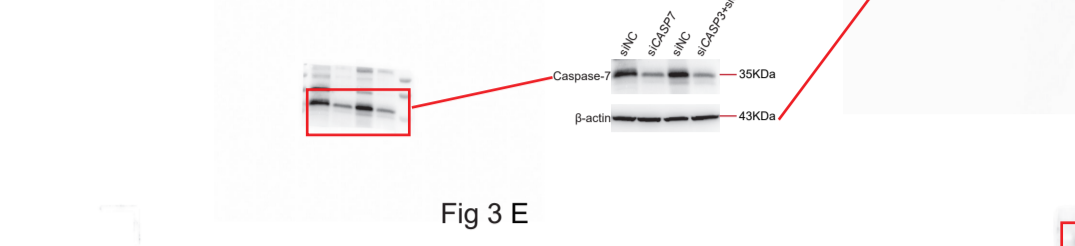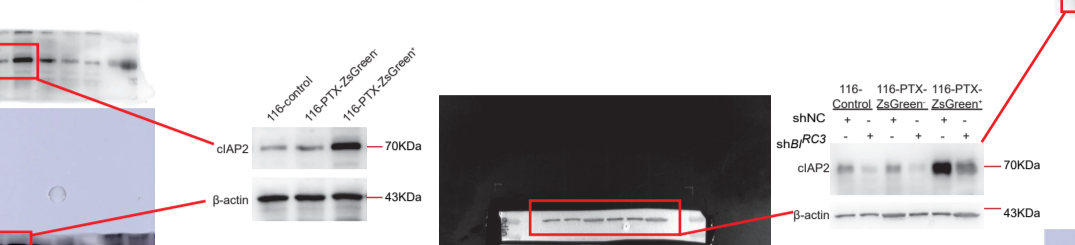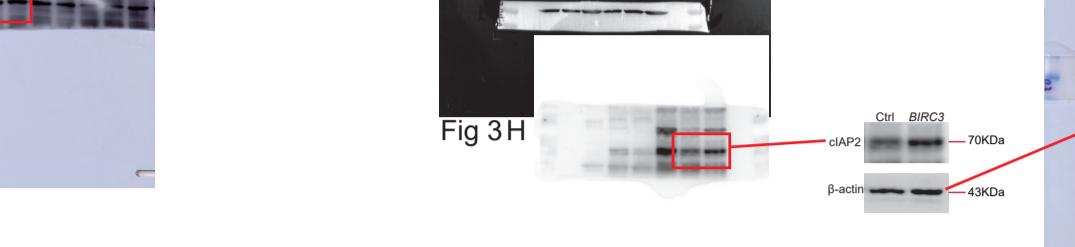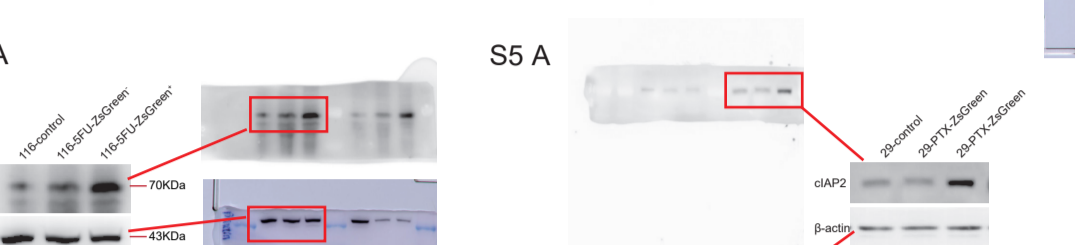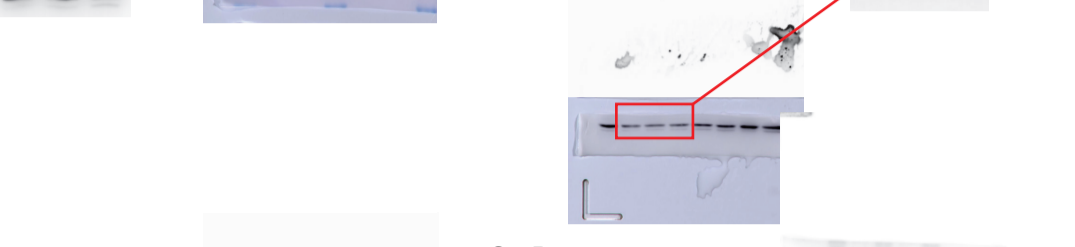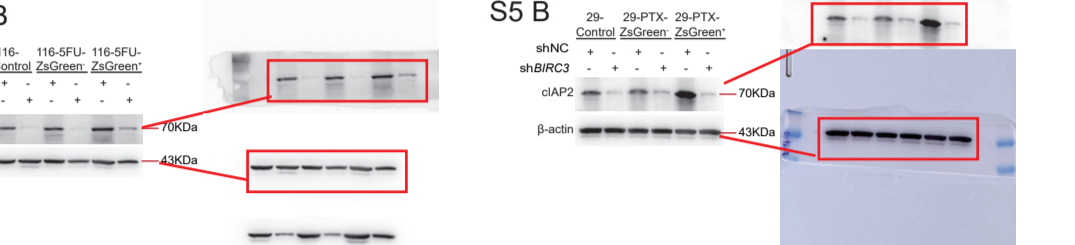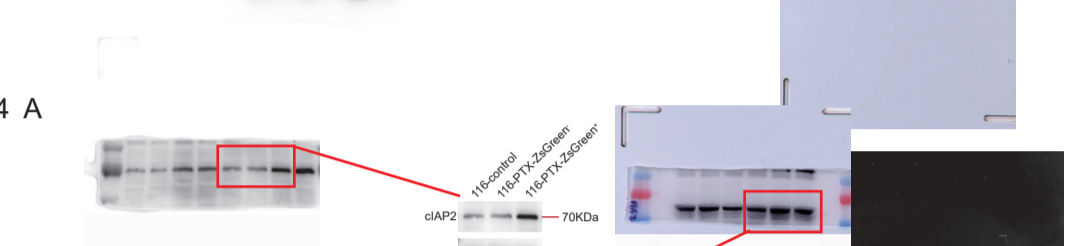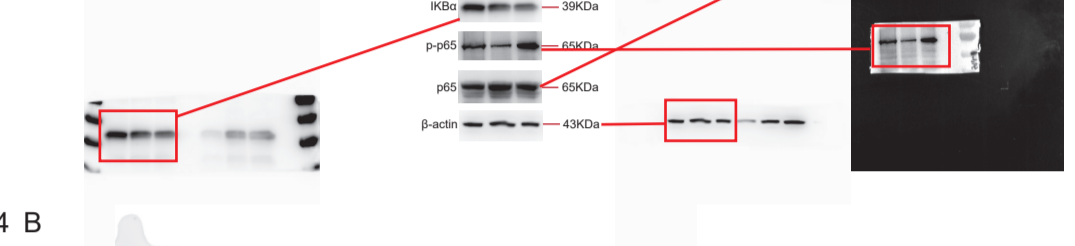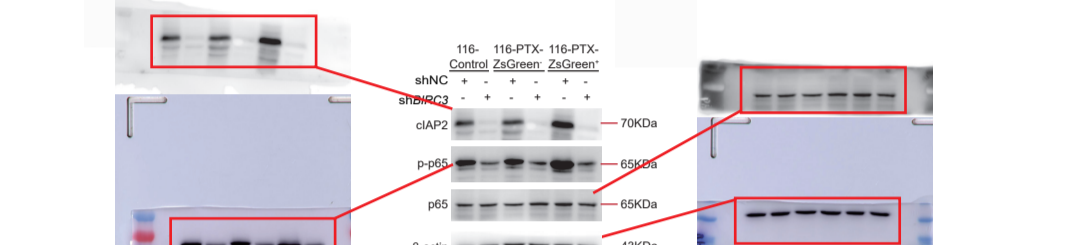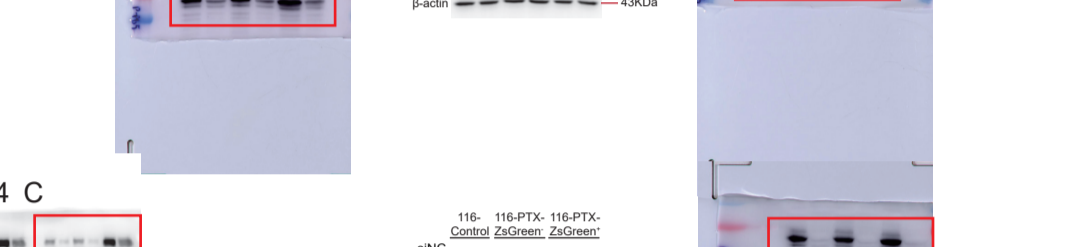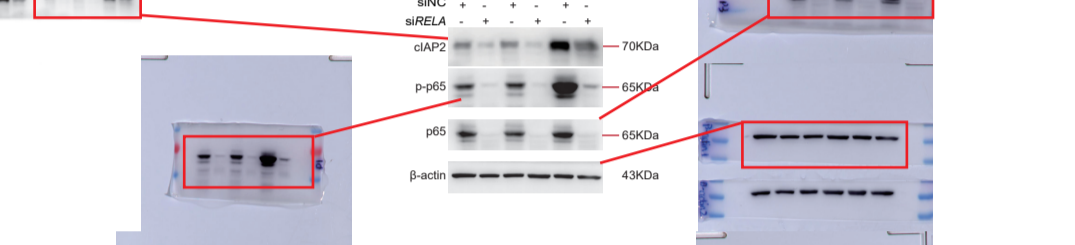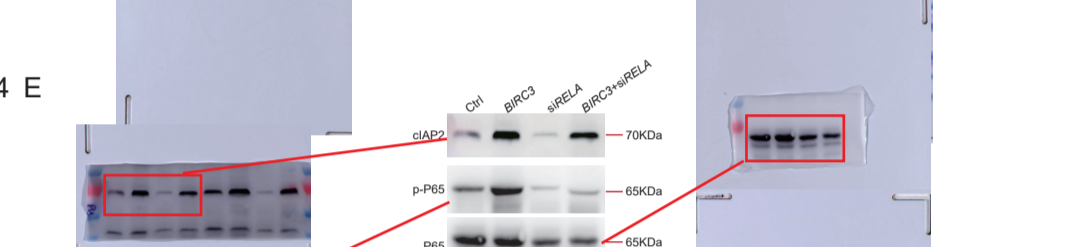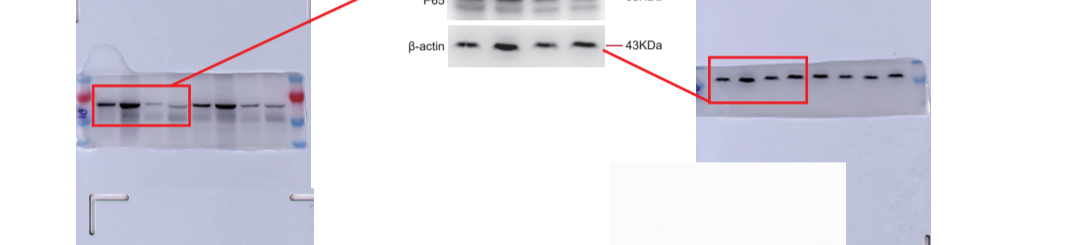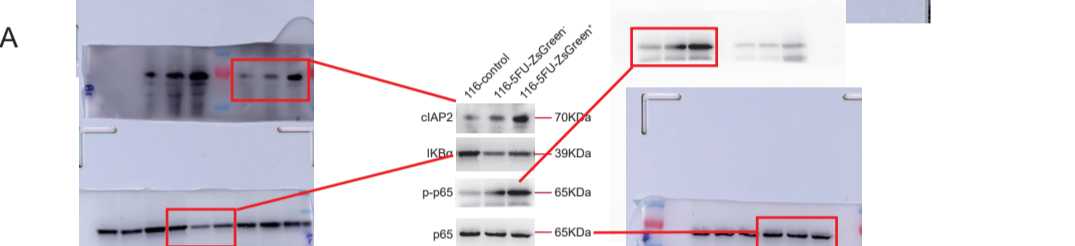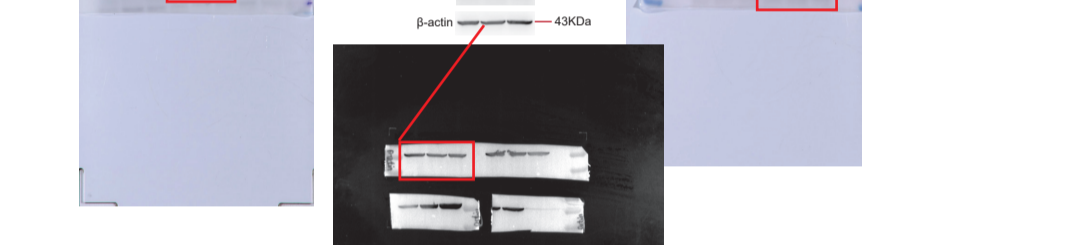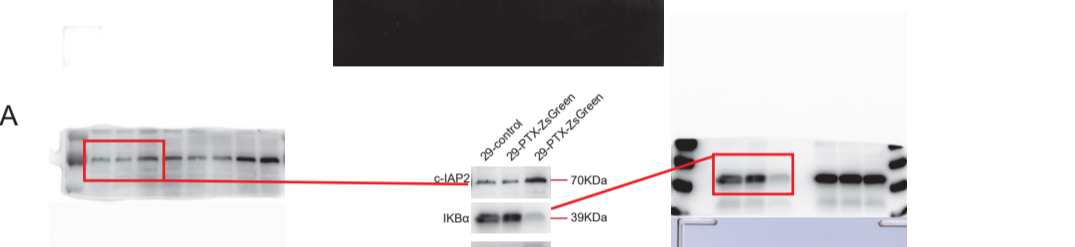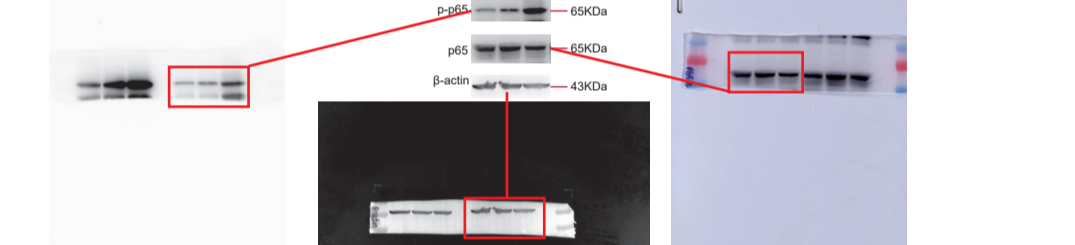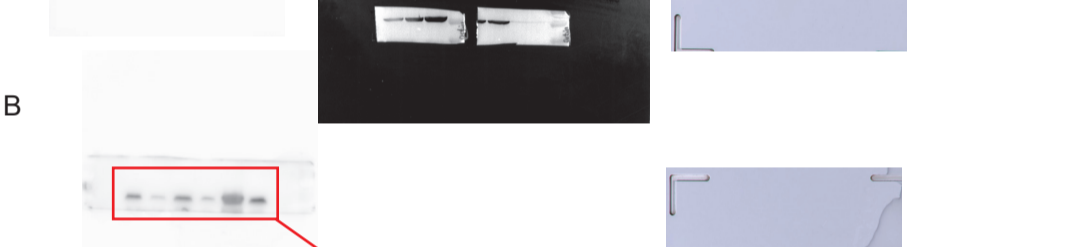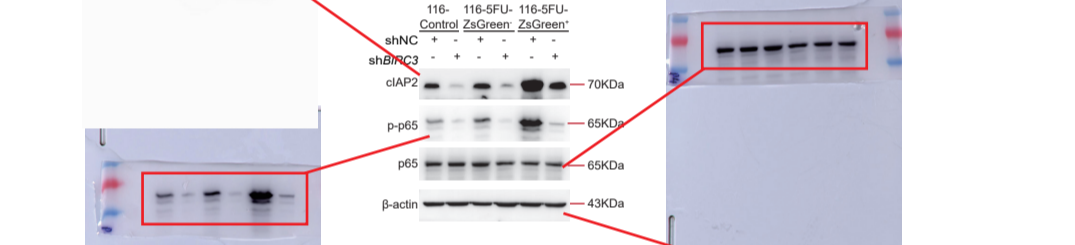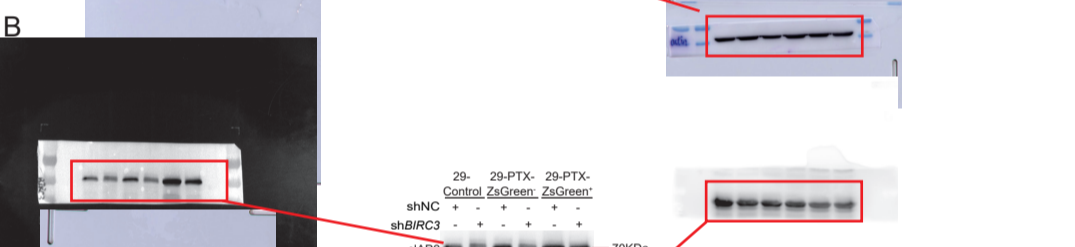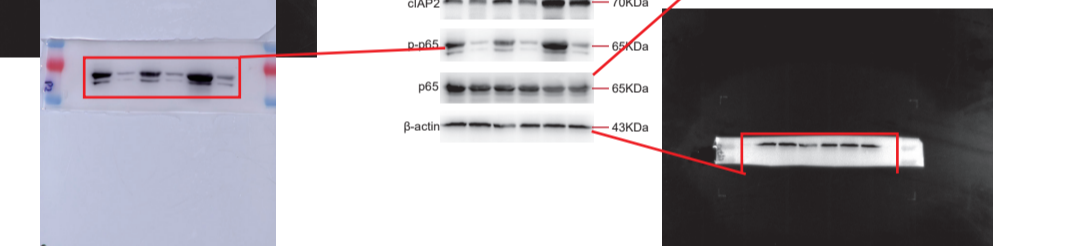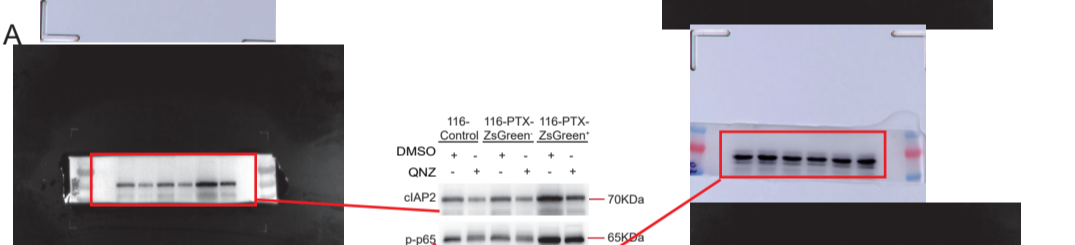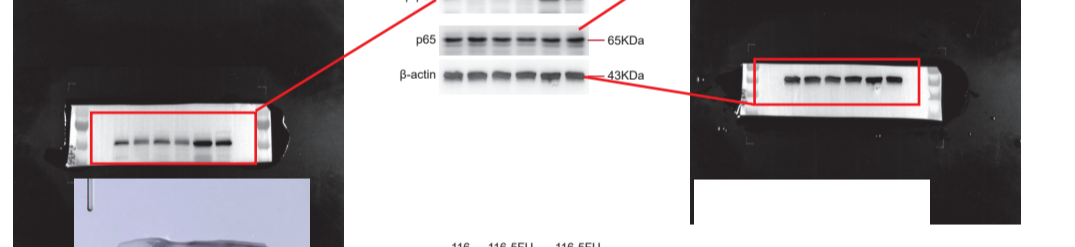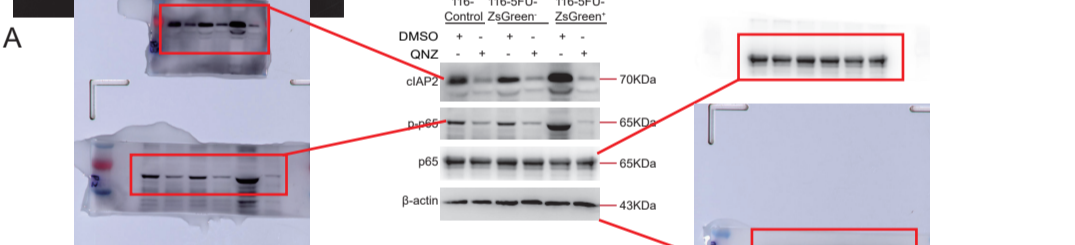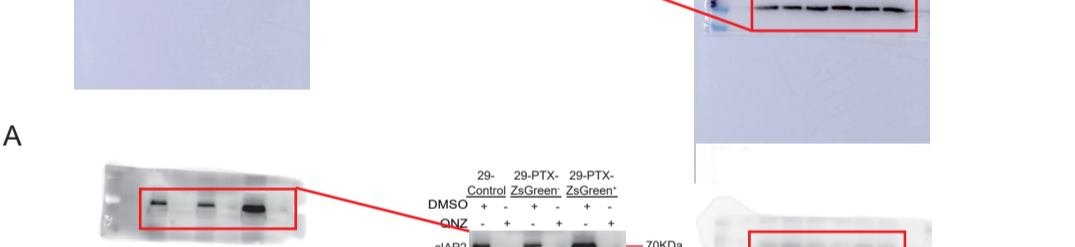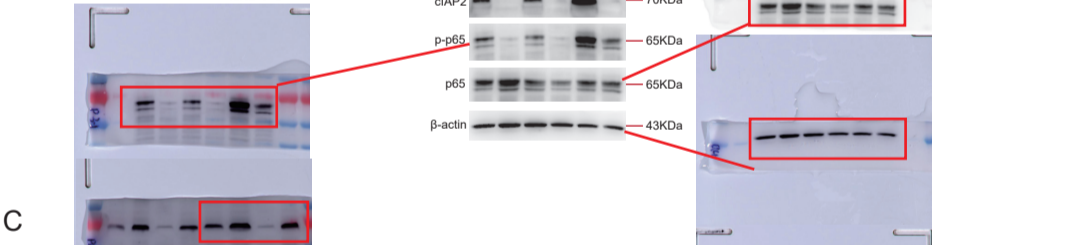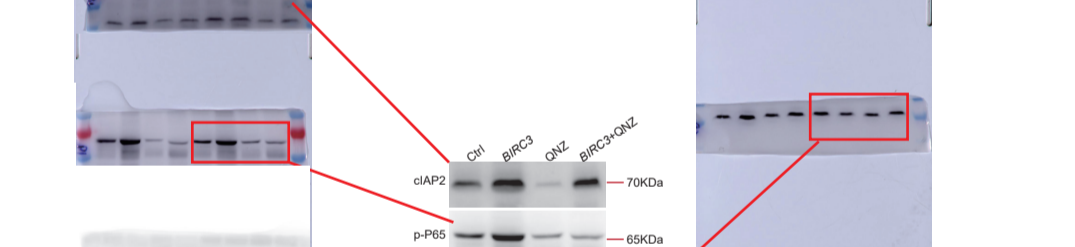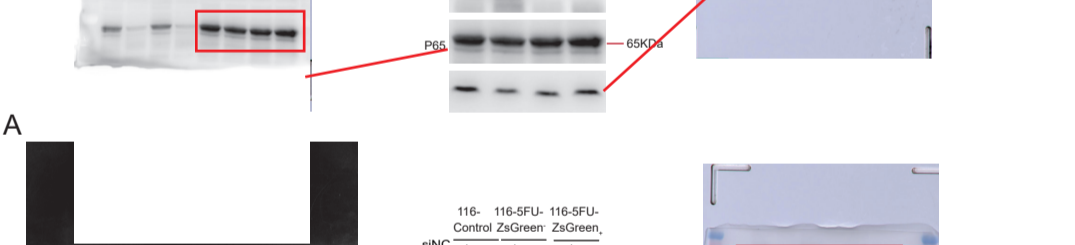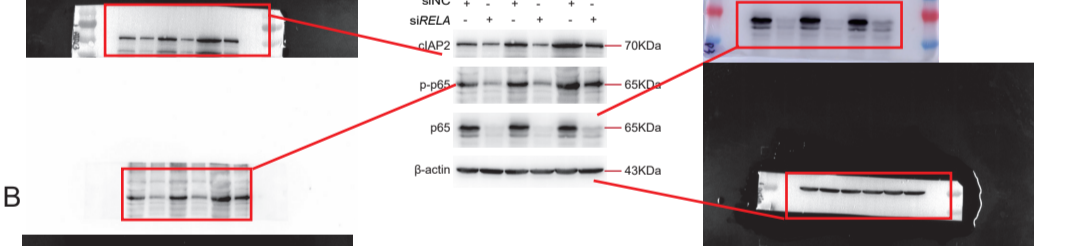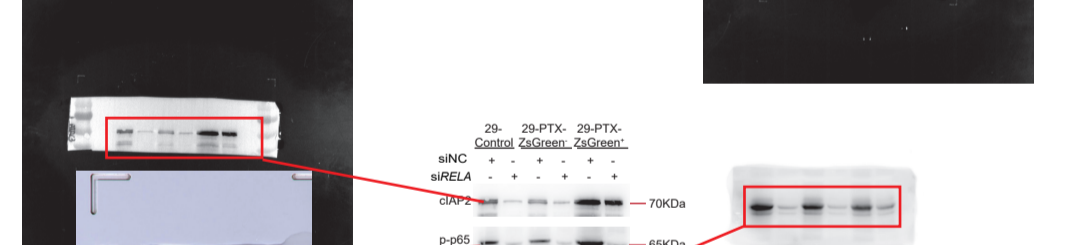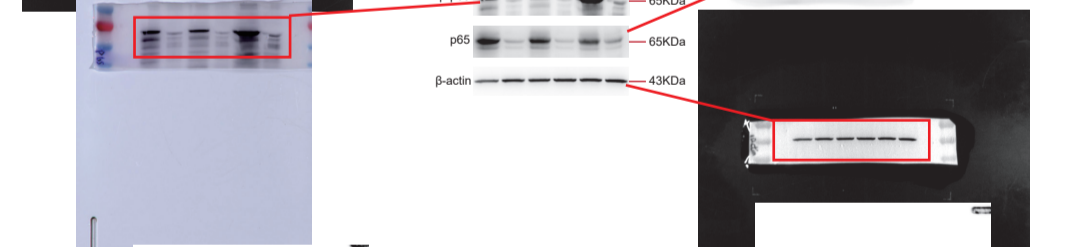

Supplement: Supplementary file 3 — Original Western blots [file 41419_2023_5916_MOESM3_ESM.pdf]
